# Anticipating the Next Recorded Bacterial Hosts of Plasmid-Borne Antimicrobial Resistance Genes: A Temporal Graph Analysis of Plasmid and Surveillance Data

**DOI:** 10.3390/genes17101291

**Published:** 2026-10-15

**Authors:** Waad A. Aljohani

**Affiliations:** Department of Basic Health Sciences, College of Applied Medical Sciences, Qassim University, Buraydah 51452, Saudi Arabia; wa.aljohani@qu.edu.sa

**Keywords:** antimicrobial resistance, horizontal gene transfer, plasmids, genomic surveillance, mobile genetic elements, molecular epidemiology, bacterial host range

## Abstract

Background/Objectives: Plasmid-mediated dissemination of antimicrobial resistance genes complicates the control of bacterial infections. Identifying bacterial hosts likely to harbour these genes could guide genomic surveillance before such associations enter public databases. We investigated whether existing plasmid and surveillance data could anticipate which resistance gene–host associations would next be recorded in those databases, and which genomic features inform that prediction. Methods: We integrated 72,556 complete plasmid records and 2,116,241 surveillance isolates, encompassing 458 resistance gene alleles, 574 replicon backbones and 222 bacterial species. A temporal graph model, MobiGraph, was trained on associations recorded before a fixed cutoff and evaluated on subsequently observed associations, using candidate hosts matched for sequencing effort and phylogenetic distance. Ranking within a candidate pool was the primary outcome; threshold-free summaries are reported as secondary. Results: The observed host ranked among the ten highest-priority candidates in 42.4% of test cases; the advantage over gradient boosting on mechanistic features was confined to the ranking metrics, the two being indistinguishable on area under the precision–recall curve. Plasmid incompatibility-group compatibility, previous gene–host associations and shared relaxase classes were prominent predictive features. Across five resistance determinants, including mcr-1, blaNDM and tet(X4), 43–62% of subsequent associations were ranked within the top ten. Among recovered events, the median interval from the prediction cutoff to first recorded observation was 22.5 months. In a separate evaluation of a fixed 500-entry watch list, 65 entries were subsequently recorded as gene–host pairs over 25 months, 13.0% against an 8.2% background rate (95% CI 10.3–16.2%); because the surveillance database does not resolve the replicon, this confirmation does not extend to the predicted backbone. Conclusions: Existing plasmid and genomic surveillance records contain information that can help anticipate which resistance gene–host associations will next be recorded. Whether a newly recorded association reflects recent transfer or earlier under-sampling cannot be resolved from these data. These findings support prioritising bacterial hosts for targeted surveillance and experimental investigation of plasmid-mediated resistance dissemination.

## 1. Introduction

Colistin resistance conferred by mcr-1 was described in *Escherichia coli* from Chinese livestock in late 2015, and within months it had been reported from human infections on four continents [1,2]. The determinant itself was not new. What had changed was its genetic context: a small plasmid that transfers readily between enterobacteria and carried the gene into species not previously under colistin selection. Comparable transitions have followed the same course. The blaNDM carbapenemases spread across *Enterobacterales* on a handful of backbones after their first description [3,4]. Tigecycline resistance mediated by tet(X4) travelled through *Escherichia* and *Acinetobacter* [5,6], optrA through enterococci [7], and cfr through staphylococci and enterococci [8]. In each case the determinant already existed in some reservoir. What changed was the replicon backbone carrying it and the host species in which it was found [9,10,11,12]. Against the burden of 4.95 million deaths associated with bacterial antimicrobial resistance in 2019, of which 1.27 million were directly attributable [13], combined with a 2021 estimate of 1.14 million attributable deaths with continued growth projected to 2050 [14], these transitions are the events that matter clinically, with each described only after it had occurred. Two developments now make it reasonable to ask whether they can instead be anticipated. Genomic surveillance has become an infrastructure rather than a series of studies: national and international programmes deposit hundreds of thousands of dated, geolocated isolates each year, genotyped through a common resistance-gene catalogue [15,16,17,18], and curated plasmid databases record which backbones carry which cargo in which organism, with mobility predicted from the sequence itself [19,20,21]. The movement of a resistance gene between hosts therefore leaves a dated trail in the public archives. The material a forecast would need has already been deposited, which raises the question of what has so far been asked of it.

Two lines of computational work have used this material well. The first reconstructs where genes have gone. Ellabaan and colleagues identified mobilisation elements statistically and predicted the phylogenetic reach of known resistance genes, confirming a subset in an independent genome collection [22]; function-based methods predict which bacterial pairs are likely to have exchanged genes from their functional content, and report discrimination close to perfect [23]. The second predicts resistance phenotypes from a finished genome and has become dependable at it [24,25,26]. Both are mature, and neither is aimed at the question a surveillance programme actually faces. Knowing that a gene could in principle reach a genus does not tell us which organism to watch. Knowing what an isolate can already resist serves treatment, not anticipation. The forward question—given this gene on this backbone, in which host will the combination next be recorded, and how far ahead can that be said—has not, to our knowledge, been posed as a prediction problem and tested on data the model had not seen. Posing it that way is more demanding than it first appears, because three things must hold at once. Edges must carry the date on which the isolate was collected, so that a model can be trained on the past and scored on the future rather than on edges censored at random from a finished network, which is the standard and considerably more forgiving protocol in link prediction [27,28,29]. The alternatives a prediction is scored against must be comparable to the true answer in how heavily they have been sequenced, since the deposition record is concentrated in a few species, countries and years, and a model compared against arbitrary alternatives can score well by learning where the sequencers are rather than how genes move [30,31]. And a forecast handed to someone with a finite budget for follow-up needs an error rate attached, which the standard distribution-free tools supply only when calibration and deployment data are exchangeable—an assumption a model trained on the past and used on the future does not satisfy [32,33]. Each requirement has an established solution in its own literature. They have not been brought together on this problem.

Here, we ask whether the host in which a resistance gene will next be recorded can be named before that record appears. We treat the problem as link prediction in continuous time on a heterogeneous graph whose nodes are resistance gene alleles, replicon backbones and host taxa, and whose edges carry the collection date of the isolate in which they were seen, assembled from 72,556 complete plasmid records and 2,116,241 surveillance isolates. The quantity predicted is the first database record of a gene–backbone–host combination. That is an observation about what surveillance has reported, not a direct measurement of transfer, stable establishment or epidemiological spread, and the distinction is kept explicit throughout. Models are trained only on associations first recorded before a fixed cutoff and scored on those first recorded after it, against candidate hosts matched on sequencing effort and phylogenetic distance, with predictions wrapped in a conformal layer calibrated under the covariate shift a temporal split creates. The aim is a shortlist that names a gene, a backbone and a host, states how far ahead the prediction is made, and carries an error rate that holds across the boundary between the data the model learned from and the data it is applied to.

## 2. Materials and Methods

### 2.1. Data Sources

The replicon layer came from PLSDB, release 2024_05_31_v2, which contains 72,556 complete bacterial plasmid records with per-record annotation from MOB-suite, PlasmidFinder, AMRFinderPlus and the Resistance Gene Identifier [19,20]. Resistance gene calls, replicon typing and mobility prediction were taken from that release rather than recomputed, so node definitions match what a user of the database obtains. AMRFinderPlus calls are the primary resistance gene annotation throughout. The Resistance Gene Identifier calls distributed in the same release were used only to assess concordance between callers and were never merged into the graph, so no node identity depends on reconciling the two. The surveillance layer came from NCBI Pathogen Detection, using isolate metadata tables for eight organism groups: *E. coli*/*Shigella*, *Salmonella*, *Klebsiella*, *Acinetobacter*, *Enterococcus faecium*, *Staphylococcus aureus*, *Pseudomonas aeruginosa* and *Campylobacter* [16]. These tables supplied 2,116,241 isolates with collection dates, geographic origin and AMRFinderPlus genotype calls, and were used to estimate sequencing effort per host taxon and to recover collection dates for plasmid records. Isolate collection dates span 1 July 1951 to 1 July 2024 in the plasmid layer, which ends at the corpus cutoff, and 1 January 1950 to 28 July 2026 in the surveillance layer, where 98% of isolates were collected between 2001 and 2026. Every source was accessed on 7 August 2026, and the Pathogen Detection snapshot identifiers for all eight organism groups are recorded in the version manifest. Reference layers were the AMRFinderPlus Reference Gene Catalog and Reference Gene Hierarchy, database version 2026-05-15.1 [17,18]; the Comprehensive Antibiotic Resistance Database [34]; and the Genome Taxonomy Database release 232, supplying the standardised bacterial taxonomy and the bac120 reference tree [35,36]. Every downloaded file was checksummed and release identifiers were recorded in a version manifest.

### 2.2. Recovering Collection Dates

PLSDB distributes NUCCORE_CreateDate, the date on which NCBI created the record, and not the date the isolate was collected. Deposition lags collection by months to years, though the lag has shortened over the period the database covers, so we rebuilt the collection date from primary sources. Each plasmid record was linked to its BioSample accession. Where that accession appeared in the Pathogen Detection isolate tables, we took the collection date parsed from that source. For the remainder we queried the NCBI BioSample database directly and parsed the collection_date, geo_loc_name and isolation_source attributes. Records resolved by neither route fall back to the record creation date, and every record carries a label recording which of the three routes supplied its date. Dates arrive at three resolutions. A year-only date was placed at 1 July and a year–month date on the fifteenth of that month. Dates before 1950 or after the date of access were set to missing.

### 2.3. Graph Construction

Gene mobilisation was represented as a heterogeneous graph in continuous time with four node types and six relation types, each edge carrying the collection date of the isolate in which it was observed. Antibiotic resistance gene nodes are allele-level symbols as called by AMRFinderPlus, for example, blaNDM-5 rather than blaNDM. Calls whose drug class was a metal, biocide or general stress determinant were removed. A prevalence rule then removed genes belonging to a species core genome: for any species with at least 200 isolates, an allele present in at least 95% of them was treated as intrinsic to that species. Twenty alleles met this criterion in every species carrying them and were excluded, removing 2,357,052 of 13,215,338 calls. The full prevalence table is released with the code. Replicon nodes are MOB-suite primary backbone clusters rather than individual plasmid records [21,37]. Backbone clusters recur across years, hosts and countries, and are the level at which a surveillance programme acts. Host nodes are bacterial species. Species strings were normalised to a binomial and matched to the Genome Taxonomy Database, which splits polyphyletic genera with an alphabetic suffix, so matching used a suffix-stripped key as well as the exact name. Mobile element nodes are the marker families the MOB-suite reports for each record: replicon types, relaxase families, mate-pair-formation systems and origins of transfer. The six relations are an ARG carried on a backbone; a backbone residing in a host; an ARG present in a host, derived from the first two and used as auxiliary supervision; an ARG flanked by a mobile element on the same replicon; two hosts closer than 0.05 patristic units on the GTDB tree; and two backbones whose member records fall below the PLSDB Mash distance threshold [38]. Records that PLSDB flags as sequence-identical were collapsed to their earliest member. Alleles observed on fewer than five records, host species carrying fewer than three records and backbones seen on fewer than three records were dropped. The resulting graph contains 458 ARG alleles, 574 backbones, 222 host species and 285 mobile element families, joined by 731,686 timestamped edges over 99,910 observations. Each triple was assigned the earliest date at which it was observed anywhere.

### 2.4. Patristic Distances

Distances between host species were computed on the GTDB bac120 reference tree. Each corpus species was mapped to a species-representative accession present on the tree, and distances were obtained from root-path sums with an ancestor-matching lowest common ancestor step. Of 222 species, 178 are on the tree. Pairs with at least one member absent received a rank-based distance calibrated to the branch lengths typical of that rank, which applied to 35.8% of pairs.

### 2.5. The Mechanistic Feature Block

Each candidate triple carries a 24-dimensional vector of hand-built mechanistic quantities, denoted psi. It contains the predicted mobility class of the backbone as three indicators; detection of an origin of transfer; detection of a mate-pair-formation system; backbone length and guanine-cytosine content; the guanine-cytosine difference between backbone and host; whether the host already carries a replicon of the same incompatibility group; whether the host already carries a replicon of the same relaxase class; the patristic distance from the candidate host to the nearest host already carrying the gene and to the centroid of that gene’s host range; logarithms of deposition volume, gene prevalence and backbone prevalence; the number of hosts and backbones the gene has been observed on; logarithms of how often the gene family has co-occurred with this incompatibility group and with this host genus; an indicator for whether the host is new for the gene; and the genomic distance in kilobases from the gene to the nearest relaxase, origin of transfer, mate-pair-formation marker and replicon marker on the same molecule. The four genomic distances were computed from the marker coordinates PLSDB distributes and aggregated to the level of a gene on a backbone by taking the median across member records. Where a replicon carries no marker of a given class the distance was set to 500 kilobases, which exceeds the largest plasmid in the corpus. Every quantity in psi was accumulated strictly from data at or before the training cutoff, and each element was standardised using training-window means and standard deviations.

### 2.6. Effort-Matched Negative Sampling

Negatives were drawn from a pool matched on sequencing effort and phylogenetic distance. For an observed triple consisting of gene *a*, backbone *r* and host *h* at time *t*, we held the gene and the backbone fixed and varied the host, which is the decision a surveillance programme faces. A candidate host qualified as a negative when it satisfied three conditions: it already existed in the graph at time *t*, so the comparison is one a contemporaneous observer could have made; it fell in the same bin of deposition effort as the true host, where effort is the base-ten logarithm of the number of genomes deposited for that taxon before the cutoff and bins are sextiles of that quantity; and it fell in the same bin of minimum patristic distance to the set of hosts already carrying the gene, binned into sextiles. Let H(*t*) be the set of host taxa present in the graph at time *t*, B_E_(*h*) the sextile of the base-ten logarithm of genomes deposited for host *h* before the cutoff, and B_D_(*h*, *a*, *t*) the sextile of the smallest patristic distance from *h* to any host carrying gene *a* at time *t*. The negative pool is

N(*a*, *r*, *h*, *t*) = {*h*′ ∈ H(*t*)∖{*h*}: B_E_(*h*′) = B_E_(*h*), B_D_(*h*′, *a*, *t*) = B_D_(*h*, *a*, *t*)} (1)

where the joint match returned an empty pool, the constraint on phylogenetic distance was relaxed and the effort match retained, and the frequency of relaxation was recorded. Balance was assessed by the standardised mean difference in deposition effort between true and sampled hosts [39,40]. A second sampler drew the negative host uniformly from all hosts present at the event time, and both samplers were applied to identical positives. Each pool was filled to one hundred draws. Where the matched pool held fewer than one hundred distinct hosts, which is usual, candidates were drawn with replacement, so a pool spans a median of 17 distinct alternative hosts (interquartile range 13 to 21). Recall at depth 100 is therefore close to saturation for any method by construction and is reported in the Supplementary Material rather than in the main tables.

### 2.7. Splits

Four protocols were used, all defined on first-observation dates. The temporal split trains on triples first observed on or before 31 December 2020, validates on those first observed during 2021 and tests on those first observed afterwards, yielding 16,064 training, 1665 validation and 2443 test triples. The inductive split is the subset of the test set in which at least one of the three endpoints was unseen before the cutoff, containing 60 queries, or 2.5% of the test set. The rolling-origin protocol repeats the temporal split at cutoffs spaced six months apart. The geographic holdout assigns each triple to the region where it was first observed and evaluates European and non-European triples separately; assignment by first observation prevents a triple that later spreads worldwide from leaking the held-out region into training. Node identities were taken from the whole corpus, since the inductive split requires post-cutoff nodes to exist in the index. Node features were accumulated strictly from the training window, and entities first observed afterwards received the zero vector [41].

### 2.8. Model

The encoder is a heterogeneous graph network in continuous time. Each node carries a memory vector updated by a gated recurrent unit whenever an event touches it. For an event between nodes *u* and *v* at time *t*, the message is a function of both memories, the elapsed time since the source was last updated, and an embedding of the relation type. Elapsed time enters through Bochner features, the cosine of a learned linear function of the time gap, with frequencies initialised on a geometric grid spanning days to a decade. Messages are mechanism-typed. Conjugation, mobilisation in trans and transposition have different kinetics and different barriers [11,42,43], so the route was treated as a latent categorical variable with a posterior inferred from the mechanistic annotation attached to the edge. The message is the posterior-weighted mixture of mechanism-specific message functions:

(2) $$m\left(u,v,t\right)=\sum_{k=1}^{K}q_{k}\left(\psi_{\mathrm{uv}}\right)\cdot f_{k}\left(s_{u}\left(t^{-}\right),s_{v}\left(t^{-}\right),\varphi \left(\Delta t\right),e_{\mathrm{uv}}\right)$$

where *s_u_*(*t*^−^) is the memory of node *u* immediately before the event, *φ*(Δ*t*) the encoded time gap, *e_uv_* the edge features, *q_k_* the posterior probability of mechanism *k*, and K the number of mechanisms, here four. The posterior is produced by a small network over the mechanistic features with a softmax temperature above one, which keeps every message function receiving gradient during early training. When the annotation is uninformative the posterior remains near uniform and the layer reduces to typed message passing. Embeddings are formed by relation-aware temporal attention over each node’s twenty most recent neighbours, held in a ring buffer written during chronological replay. Query, key and value projections are indexed by relation. The decoder scores a triple. It concatenates the three time-aware embeddings and their three pairwise elementwise products, compresses that to 64 dimensions with a linear bottleneck, appends psi unchanged, and passes the result to a Kolmogorov–Arnold network [44], in which every connection is a learned univariate spline. Passing psi through unchanged preserves a separate spline function for each named mechanistic quantity. Training replays observations in strict timestamp order. Each batch is scored using the memory as it stood immediately before those events, and the memory absorbs them only afterwards. The objective combines four terms:

L = L_rank_ + *λ*_cal_L_cal_ + *λ*_aux_L_aux_ + *λ*_reg_L_reg_ (3)

The ranking term is a pairwise logistic loss requiring each observed triple to outscore its own matched negatives. The calibration term is a focal cross-entropy with label smoothing. The auxiliary term regresses the time elapsed since the gene’s memory was last updated. The sparsity term acts on the scoring head. Let *φ_ij_* denote the mean absolute activation of the spline connection from input *i* to output *j*:

(4) Lreg=∑ijφij−∑ijpij log pij

where the normalised activation share is

(5) $$p_{\mathrm{ij}}=\frac{\left|\varphi_{\mathrm{ij}}\right|}{\sum_{\mathrm{kl}}\left|\varphi_{\mathrm{kl}}\right|}$$

The first part shrinks all connections, and subtracting the entropy of the normalised activations concentrates the surviving activation onto few connections. Hyperparameters were fixed across all arms: memory dimension 128, four attention heads, twenty buffered neighbours, twenty negatives per positive during training, batch size 512, AdamW at learning rate 1 × 10^−3^ with weight decay 1 × 10^−5^, gradient clipping at 5, and early stopping on validation area under the precision–recall curve with patience 5. Weights on the three auxiliary terms were 0.30, 0.10 and 1 × 10^−4^. The full model has 842,629 parameters and trains in under seven minutes on one consumer graphics card.

### 2.9. Baselines

Every baseline received the same positives, the same negatives and the same features, with candidate construction implemented once and shared. Tier one comprises popularity, which scores a candidate by how often the host appears in the training window; Adamic-Adar and Jaccard on the static gene–host projection [27,45]; a hand-written rule scoring a candidate by whether the host already carries a replicon of the same incompatibility group and the same relaxase class; and EdgeBank, which returns one if the pair has been seen and zero otherwise [29]. Tier two comprises a statistical mobilisation-reach heuristic in the spirit of earlier work, scoring a candidate host by how far inside the gene’s observed phylogenetic radius it sits, scaled by the fraction of that gene’s observations falling on a mobilisable or conjugative backbone [22]; gradient boosting fitted on psi alone with no graph information [46]; and implicit-feedback matrix factorisation with alternating least squares on the gene-by-host matrix. Tier three comprises graph convolutional, GraphSAGE, graph attention, relational graph convolutional and heterogeneous graph transformer architectures for the static case, together with continuous-time models in the style of JODIE and DyRep and sequence-based temporal encoders [28,47,48,49,50].

### 2.10. Ablations

Eight arms were trained under the identical protocol: removing psi; removing the time encoding; removing the host phylogeny features; reducing node features to the type indicator; sharing one weight set across all relations; replacing the Kolmogorov–Arnold head with a multilayer perceptron of comparable capacity; removing the latent-mechanism mixture; and training against uniformly drawn negatives. The full model was trained at three seeds and each ablation at one seed.

### 2.11. Evaluation

Ranking metrics were computed within each candidate’s own matched pool, containing one true host and one hundred matched alternatives. Because a surveillance programme orders candidates inside a pool and acts on the first few, the primary outcomes are the mean reciprocal rank and recall at depths 1, 5 and 10. Area under the precision–recall curve and under the receiver operating characteristic curve are reported as secondary, threshold-free summaries [51,52,53,54]. Calibration was summarised by expected and maximum calibration error over quantile bins and by the Brier score [55,56,57,58,59,60,61]; quantile rather than equal-width bins were used because equal-width bins place almost every candidate in the first bin at this prevalence. Percentile bootstrap intervals over queries, with 2000 resamples, accompany every ranking metric, and Wilson intervals are used for proportions estimated on small strata. Differences between the two methods were assessed with a bootstrap paired at the level of the query, since the two runs share the positives and the matching protocol but draw their own candidate sets [62]. Lead time is the interval between the training cutoff and the first observation of a true triple, computed for triples the model placed in the top k of their pool at the cutoff, with triples never reaching that depth counted as misses.

### 2.12. Conformal Risk Control

Split conformal prediction provides distribution-free guarantees when calibration and test data are exchangeable [63,64,65,66]. Two arms were run. The exchangeable arm splits the post-cutoff window into two random halves by query. The temporal arm calibrates on the validation year and evaluates on the later test years. Three procedures were compared. Naive split conformal takes the empirical quantile of the calibration scores. Weighted conformal prediction reweights those scores by an estimated likelihood ratio between test and calibration covariate distributions [31,67]; the ratio was obtained from a gradient-boosted classifier trained to separate calibration-era from test-era candidates using out-of-fold probabilities, given the mechanistic features only, and clipped to the interval from one twentieth to twenty. Non-exchangeable conformal prediction applies fixed weights decaying with the age of the calibration point, with a one-year half-life [33]. Because the deliverable is a set, we controlled the false-discovery proportion of the returned shortlist rather than per-instance coverage [68]. For a score threshold λ, the shortlist is the set of candidates scoring at or above λ and the empirical risk is the proportion of that set that is negative. The risk curve was replaced by its running maximum taken from the strict end, and the empirical risk was inflated by the usual finite-sample correction before comparison with the target. Where no threshold attained the target the procedure returned the empty set and recorded that it had done so. Mondrian calibration repeated the procedure independently inside each cell of a taxonomy defined by host phylum, drug class and predicted mobility, with cells holding fewer than fifty calibration points falling back to pooled calibration.

### 2.13. Symbolic Extraction

Spline connections in the scoring head whose mean absolute activation fell below five percent of the largest in their layer were removed. Each surviving first-layer connection over a named mechanistic feature was sampled on a bounded grid and fitted with a library of elementary forms: linear, quadratic, square-root, exponential, logarithmic, logistic, hyperbolic tangent and step. The best fit by adjusted coefficient of determination was retained, and connections that no library member fitted above a floor were recorded as non-elementary. Rules were built as conjunctions of at most three threshold conditions on the highest-importance features, with thresholds placed at data quantiles. Rules selecting identical candidate sets were merged. Selection used the training window, and every retained rule was rescored as a standalone classifier on the temporal test window. Thresholds are expressed in original units [69,70,71,72].

### 2.14. Watch List

The model was frozen at the corpus cutoff of 1 July 2024, with its memory warmed on every observation to that date, and used to score unobserved triples. The candidate universe was built from the 4000 most frequent gene–backbone pairs in the graph, each paired with up to 120 host taxa lying within the eightieth percentile of the gene’s observed within-range distances and above the thirtieth percentile of deposition effort, giving 332,795 candidate triples. Mondrian conformal risk control was applied at alpha = 0.1. The 500 highest-scoring triples were written with a cryptographic digest and a freeze manifest recording the model tag, the data cutoff and the time of freezing. The freeze fixes the model, the candidate universe and the ranking on a data state ending 1 July 2024, and the file was digested before any later record was examined. The archive it is scored against already existed when the freeze was performed, so this is a blinded out-of-time validation rather than a live prospective trial; the digest is what makes rescoring against future releases genuinely prospective from here on.

### 2.15. Watch-List Confirmation

The frozen list was scored against a later NCBI Pathogen Detection snapshot. The confirmation stage verifies the digest of the frozen file before scoring and returns nothing if the digest does not match. Pathogen Detection resolves the gene and the host but not the replicon, so a candidate was counted as confirmed when its gene and host were observed together in an isolate whose collection date fell after the corpus cutoff. Confirmation is therefore at gene–host resolution only: a confirmed entry establishes that the predicted gene and the predicted host were recorded together after the cutoff and leaves the predicted backbone untested. Full triple confirmation would require a later release of a complete-plasmid database. Hit rates were computed at list depths of 10, 50, 100 and 500 and over the conformal shortlist, with Wilson intervals and one-sided binomial tests against the background rate. The confirmation window runs from the corpus cutoff of 1 July 2024 to the snapshot of 7 August 2026, a period of 25 months. The base rate for the same window was obtained by applying the identical confirmation criterion to all 332,795 candidate triples.

### 2.16. Sensitivity Analyses

Three robustness questions were put to the frozen predictions rather than to a retrained model, so that none of them could alter a headline number. Robustness to collection-date uncertainty was assessed in three ways. Test queries were stratified by the route that supplied the date and by its resolution. The evaluation was then repeated on the subsets whose dates came from a primary source and whose resolution was a day or a month. Finally, every imprecise date was resampled 500 times, with year-only dates placed uniformly inside their year, month-only dates inside their month, and records that fell back to the record creation date shifted backwards by a draw from the observed interval between collection and deposition, measured on the 49,629 records where both dates are known and with a median of 45.8 months. Each draw re-derives the first-observation date of every triple, re-derives which triples fall after the cutoff, and rescores the frozen model on the test set that draw produces.

Robustness to the choice of gene caller was assessed on the records both callers had scanned by comparing calls at allele level, at gene-family level and under a match that tolerates differences in allele-level granularity; by stratifying test queries according to whether the second caller supports the gene; and by repeating the evaluation with the gene family rather than the allele as the unit of prediction—the rank kept for a collapsed event being the best achieved by any of its member alleles. The status of the sampled alternatives was examined in the same way: every candidate host drawn for a test pool was checked against the surveillance layer to establish whether that gene–host pair had been recorded at any time, before the query date or only after it. The three analyses run as one pipeline stage, which reads the frozen prediction files and writes its result tables alongside the others.

### 2.17. Implementation and Availability

The analysis is implemented in Python 3.11 with PyTorch 2.13, NumPy 2.4, SciPy 1.18, pandas 2.3 and scikit-learn 1.9. The Kolmogorov–Arnold layers, the conformal procedures and the effort-matched sampler were written for this work. Data acquisition, graph construction, model fitting, ablation, conformal calibration, symbolic extraction, watch-list generation and confirmation run as numbered stages, and numerical results quoted in the text are substituted from the result files.

## 3. Results

### 3.1. A Dated Graph of Where Resistance Genes Have Already Travelled

**Figure 1:**
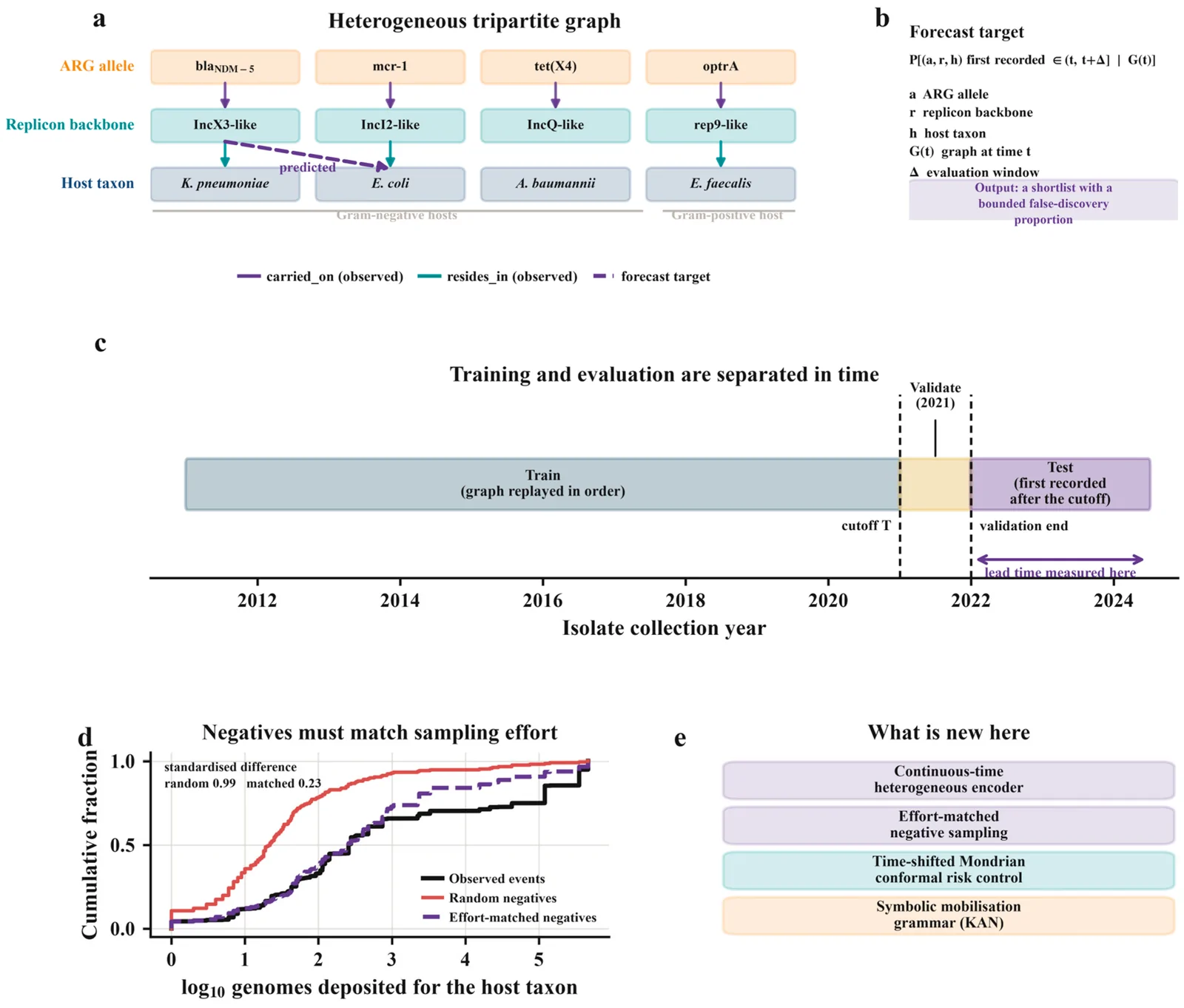
Forecasting gene mobilisation as a dated link prediction problem. (**a**) Nodes are resistance gene alleles, replicon backbones and host taxa. Gene nodes are allele-level symbols, so the carbapenemase family-written blaNDM elsewhere in the text appears here as one of its alleles, blaNDM-5. The examples span both Gram stains: optrA on an enterococcal rep9-like backbone in *Enterococcus faecalis* alongside three determinants of Gram-negative hosts. The dashed edge is the quantity to be forecast. (**b**) The predicted quantity and the form of the output. (**c**) Training, validation and test windows on the collection-date axis. (**d**) Deposition effort for observed events and for candidate hosts drawn uniformly or matched on effort as cumulative distributions over the test pools. (**e**) The four components the study assembles.

**Figure 2:**
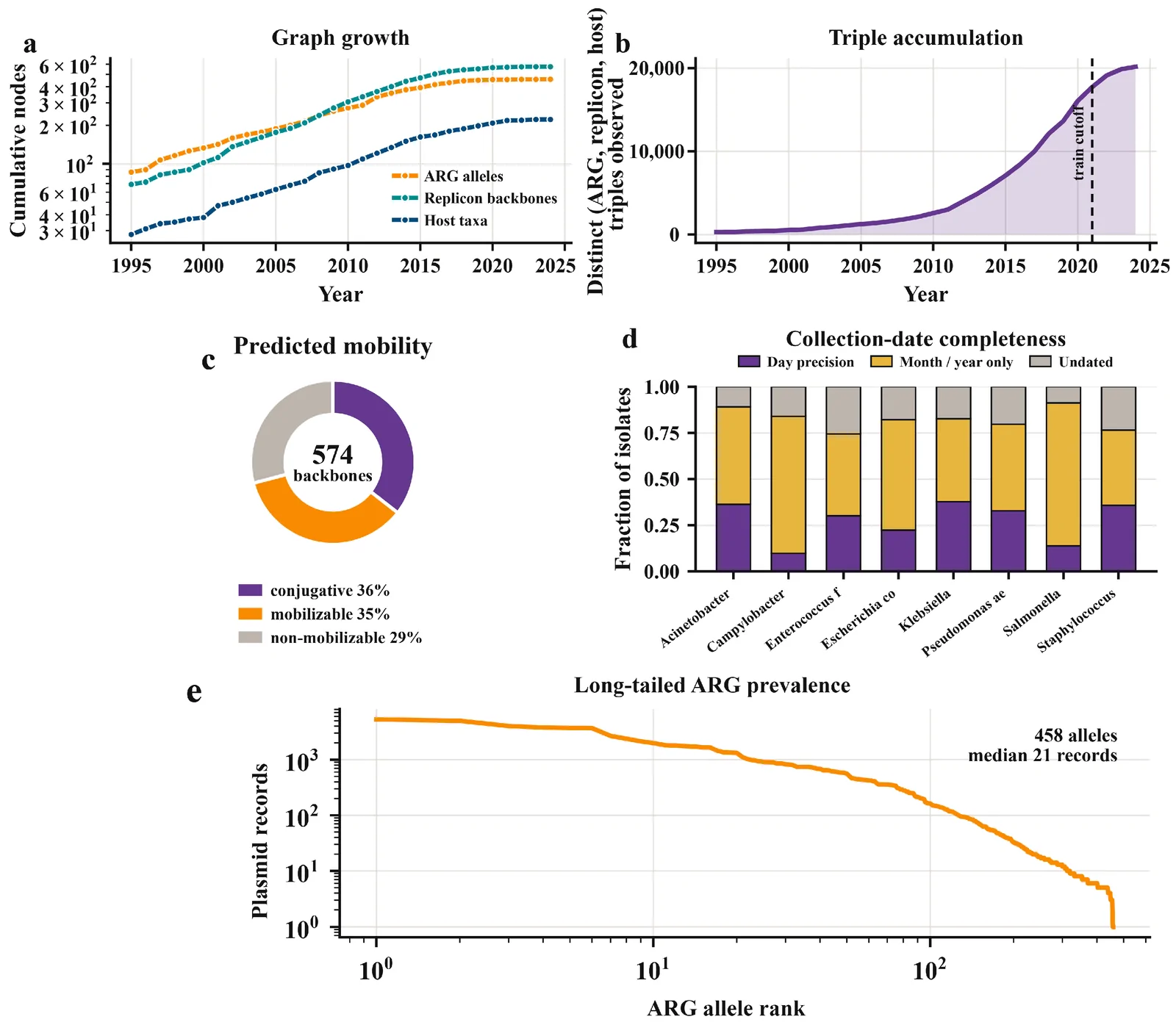
Composition of the corpus. (**a**) Cumulative numbers of resistance gene allele, replicon backbone and host taxon nodes by year (logarithmic scale). (**b**) Cumulative number of distinct gene–backbone–host triples observed; the dashed line marks the training cutoff. (**c**) Predicted mobility of the 574 replicon backbones. (**d**) Resolution of collection dates for surveillance isolates in the eight organism groups. (**e**) Number of plasmid records per resistance gene allele against allele rank (logarithmic axes).

The forecast begins with a graph in which every edge carries the date of the record behind it. The analysis has four steps: assemble that graph, train on everything recorded before a cutoff, rank candidate hosts for the associations recorded after it, and attach a calibrated error rate to the resulting shortlist (Figure 1e and Figure S1); the modelling terms used throughout are defined in Table S1. Resistance gene alleles, replicon backbones and host taxa form three node layers, joined by edges recording that an allele was annotated on a backbone or that a backbone was recovered from a taxon, each carrying the collection date of the isolate in which it was seen (Figure 1a). The quantity to be predicted is the probability that a particular gene, backbone and host are seen together for the first time within a stated window, conditioned on the graph as it stood at the moment of prediction rather than on the finished network (Figure 1b). Training therefore uses only edges first observed on or before the cutoff, and scoring uses only edges first observed afterwards, so that no information from the evaluation period reaches the model through features, hyperparameters or early stopping (Figure 1c). Because the hosts of real events have been sequenced far more deeply than hosts drawn at random, with mean log deposition 2.89 against 1.49 and a standardised mean difference of 0.98, candidate alternatives are matched on deposition effort and phylogenetic distance, which raises the negative mean to 2.53 and reduces the standardised difference to 0.23 (Figure 1d). These four elements, the dated graph, the forward split, the matched comparison and the calibrated output, are the components the study assembles (Figure 1e). Assembling the graph required recovering the timestamps, since PLSDB distributes the date each record was created at NCBI rather than the date its isolate was collected. Collection dates were recovered for 71.8% of records, 36.6% by matching BioSample accessions against the surveillance metadata and 34.5% by querying BioSample directly (Figure S2a), with 28.6% of all dates resolved to the day and the remainder to the month or year (Figure S2b); coverage improves steadily across the period the database spans (Figure S2c). Gene calls were taken from AMRFinderPlus and cross-checked against an independent caller on the 14,100 records both had scanned (Figure S3a, Table S2), and twenty species-intrinsic alleles such as blaEC, acrF and mdtM were removed by a prevalence rule so that a model could not score highly by predicting that *E. coli* still carries its own chromosomal beta-lactamase (Figure S3b). What remains is a corpus of 20,419 plasmid records carrying at least one acquired determinant, contributing 99,910 gene observations across 458 alleles, 574 backbones, 222 host species and 285 mobile element families, joined by 731,686 timestamped edges spanning 1951 to mid-2024 and 126 countries (Table 1). All three node layers accumulated slowly until the late 1990s and then steepened after 2010, when complete plasmid sequencing became routine; the host layer saturates earliest, since the taxa under surveillance are a small and stable set, while the gene and backbone layers continue to gain entities to the end of the record (Figure 2a). Roughly four fifths of all distinct gene, backbone and host combinations were already present at the training cutoff, which fixes the size of the prospective test set at 2443 triples (Figure 2b). The backbones divide into 204 conjugative, 203 mobilizable and 167 non-mobilizable replicons, so the corpus is not dominated by self-transmissible elements (Figure 2c). Collection-date completeness in the surveillance layer runs from 74.4% in *E. faecium* to 91.2% in *Salmonella* (Figure 2d), and allele frequencies are long-tailed, headed by sul1 with 5305 records, blaTEM-1 with 4988, sul2 with 4024, tet(A) with 3771, aph(6)-Id with 3704, aph(3″)-Ib with 3698, mph(A) with 2677, aadA2 with 2366, aadA1 with 2136 and floR with 1987 (Figure 2e, Table S3). Hosts are predominantly Gram-negative, which follows from what has been deposited as complete plasmids rather than from any choice made here, but the corpus is not exclusively so: 11,223 of the 99,910 observations, 11.2%, fall in *Bacillota* or *Actinomycetota*, spanning 70 species and 97 alleles, among them the oxazolidinone and phenicol determinants optrA and cfr (Figure 1a, Table S4). Deposition is concentrated: the Gini coefficient of records per backbone is 0.81 and of isolates per host taxon 0.79 (Figure S4a), and the three best-represented countries account for 60% of located records, led by China with 25,930 observations, the United States with 12,075 and South Korea with 4093 (Figure S4b). That concentration is precisely what the matched comparison is designed to hold constant.

### 3.2. Forecasting the Next Host from the Past Alone

**Figure 3:**
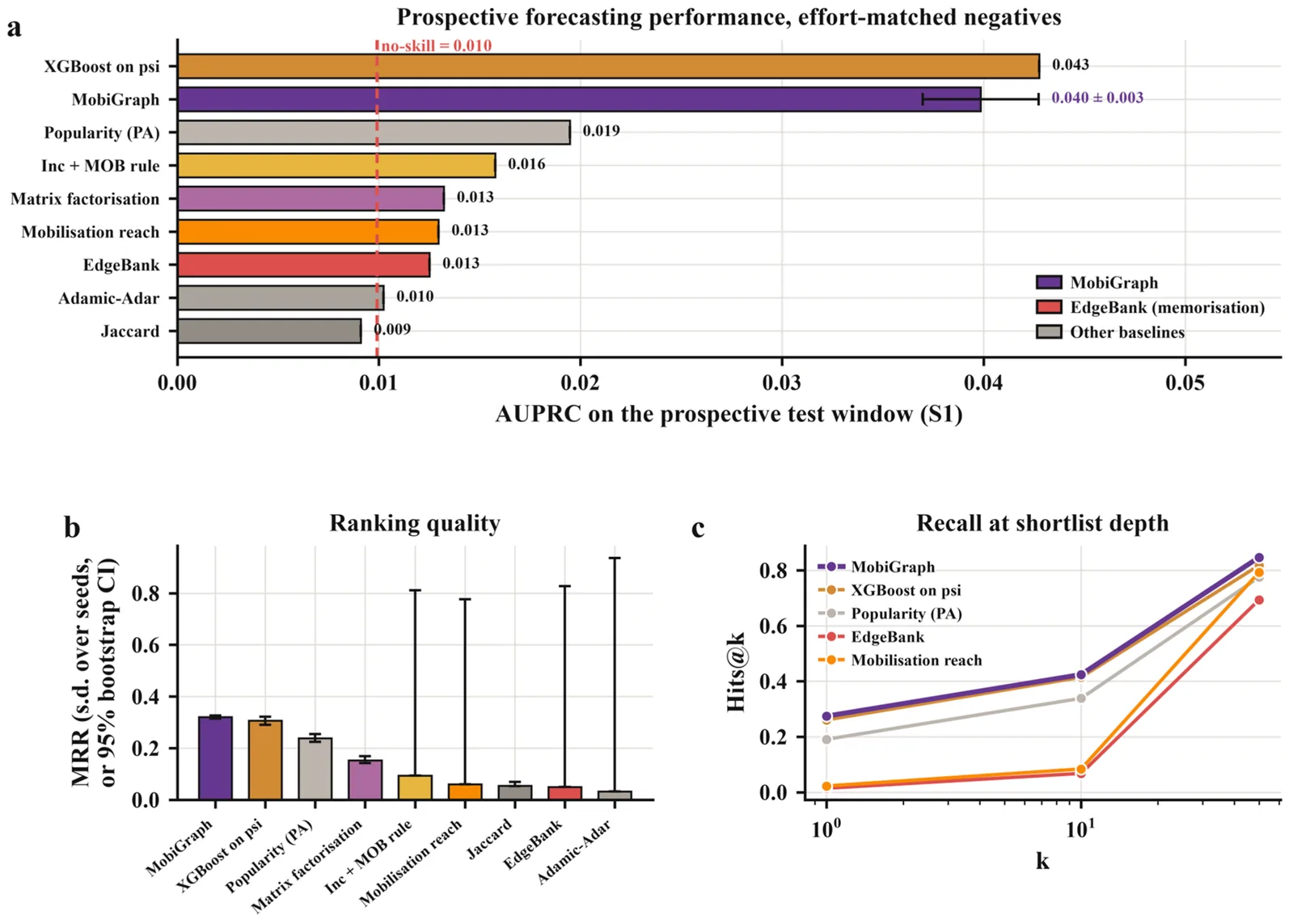
Prospective forecasting performance under effort-matched negatives. (**a**) Area under the precision–recall curve (AUPRC) on the prospective test window; the dashed line marks the no-skill level, and the MobiGraph error bar is the standard deviation across three seeds. (**b**) Mean reciprocal rank (MRR), with the standard deviation across seeds for MobiGraph and 95% bootstrap confidence intervals for the baselines. (**c**) Recall within the top k candidates (Hits@k) as a function of shortlist depth k.

Scored on triples first observed after the cutoff, against pools of one true host and one hundred effort-matched alternatives, MobiGraph (version 1.0.0) placed the true host at a mean reciprocal rank of 0.3204 with a standard deviation of 0.0053 across three seeds, ahead of gradient boosting on the mechanistic features at 0.3071 (95% CI 0.291 to 0.324), matrix factorisation at 0.1529 and the deposition-volume baseline at 0.2388 (Table 2). Area under the precision–recall curve reached 0.0399 against a positive rate of 0.0099, a fourfold lift over chance, with gradient boosting at 0.0428 and every remaining method between 0.0091 and 0.0195 (Figure 3a). On that secondary metric the two leading methods are indistinguishable: a bootstrap paired by query puts the difference at −0.00005 (95% CI −0.0091 to 0.0073, *p* = 0.91), while the same procedure separates them on reciprocal rank (0.019, 95% CI 0.007 to 0.032, *p* = 0.005). The advantage reported below is therefore an advantage in ranking, which the design treats as the primary outcome because a surveillance programme orders candidates within a pool rather than applying one global cut. On reciprocal rank the graph model led at every seed (Figure 3b), and recall curves separate most clearly between depth five and depth twenty-five, the range a triage programme would actually use, where MobiGraph recovered 27.4% of true hosts at rank one and 42.4% within the top ten (Figure 3c). EdgeBank, which returns one for any pair it has already seen and zero otherwise, reached a reciprocal rank of 0.0497, so the result is not memorisation of pairs already in the training window. The sampler balances on deposition effort and on distance to the hosts already carrying the gene (Figure S5a), and achieves standardised mean differences below the conventional threshold on both (Figure S5b). What those alternatives are is worth stating precisely, because an unobserved combination is not a verified absence. Of the 244,300 candidate hosts drawn for the test pools, 33.2% are gene–host pairs the surveillance layer has recorded at some point without a resolved plasmid, a further 0.3% were recorded only after the query date, and the remaining 66.3% have no surveillance record of the pair at all (Figure S6a). Every precision-like quantity reported here is therefore a lower bound: counting any surveillance record of the pair as a success raises precision in the top 0.1% of candidates from 15.8% to 67.6% (Figure S6b). Scoring the identical positives against uniformly drawn alternatives instead raises the area under the receiver operating characteristic curve for every method, the deposition-volume baseline from 0.6462 to 0.8469 and gradient boosting from 0.7336 to 0.8714 (Figure S7a, Table S5), with precision–recall performance rising in proportion (Figure S7b). The size of that shift runs from 1.23 for Jaccard similarity to 4.74 for the deposition-volume baseline (Figure S7c), and a baseline using nothing but deposition volume reaches 92% of the best method under uniform alternatives against 46% under matched ones (Figure S7d). The ordering of methods is identical under both protocols, with a Kendall rank correlation of 1.00, so the matched pools change what the numbers mean rather than which method wins. Eight arms were trained under the identical protocol to attribute the result to its parts (Table 3, Figure S8). The node feature block carries the most weight: reducing node features to a type indicator moved reciprocal rank from 0.3204 to 0.2741, a relative change of 14.4%. The mechanistic feature block accounted for a further 7.0%, host phylogeny for 4.7% and the time encoding for 4.0%, so each of the four inputs the design specifies is load-bearing on the ranking metric. Replacing the spline scoring head with a conventional multilayer perceptron of comparable capacity left reciprocal rank at 0.3214, which means the interpretability the spline head provides is obtained at no measurable cost in accuracy, a point the following sections depend on. Training converged within the same budget across arms (Figure S9a) with early stopping triggered on validation precision–recall performance (Figure S9b), and the full model trains in under seven minutes on a single consumer graphics card, with per-epoch and per-arm cost differing by less than a factor of two across the set (Figure S10a,b).

### 3.3. The Forecast Survives the Uncertainty in the Dates and the Choice of Gene Caller

The temporal design rests on collection dates that had to be reconstructed, so the forecast was re-examined under several treatments of that uncertainty (Figure S11, Table S8). Queries whose date came from a primary source ranked slightly better than the pooled figure rather than worse, at a reciprocal rank of 0.337 (95% CI 0.309 to 0.366) over 852 queries; restricting to records resolved to the day or the month left it unchanged at 0.319 (0.290 to 0.349) over 731 queries. Queries resting on the creation-date fallback sat at 0.312 (0.292 to 0.332), within the interval of the pooled result. Resampling every imprecise date 500 times moved the reciprocal rank between 0.326 and 0.351, with the reported 0.3204 at the lower edge of that range, and recall within the top ten between 43.7% and 47.2% against a reported 42.4%. Date uncertainty is therefore not what produces the result, though it does determine which triples fall after the cutoff: under the most aggressive backward shift only 39.9% of the original test triples remain in the test window, and the figures above are computed on whatever set each draw produces.

Gene identity enters the graph through one caller, so its influence was measured directly. On the 14,100 records scanned by both AMRFinderPlus and the Resistance Gene Identifier, 35.3% of the latter’s calls were matched symbol for symbol, rising to 51.0% once differences in allele-level granularity are tolerated, such as aadA against aadA1 on the same record. The remainder is dominated by CARD entries that AMRFinderPlus does not report as acquired determinants, including regulators such as tetR and mrx and components named for the operon they belong to, and by the two callers reporting different numbers of determinants per record, 6.7 against 2.6 (Table S9). Half of the allele nodes in the graph, 229 of 458, carry an allele-level call from the second caller and 269 carry a family-level one. The forecast does not follow that division: reciprocal rank was 0.312 (0.294 to 0.331) on queries whose gene both callers report and 0.350 (0.314 to 0.388) on the rest, intervals that overlap (Figure S12b). Repeating the whole evaluation with the gene family as the unit of prediction, which is the resolution at which the callers largely agree, gives 0.318 (0.301 to 0.336) against 0.320 at allele level (Figure S12c). The conclusions do not rest on resolving the discordance between the two catalogues.

### 3.4. Performance Holds on Entities and Regions the Model Never Saw

**Figure 4:**
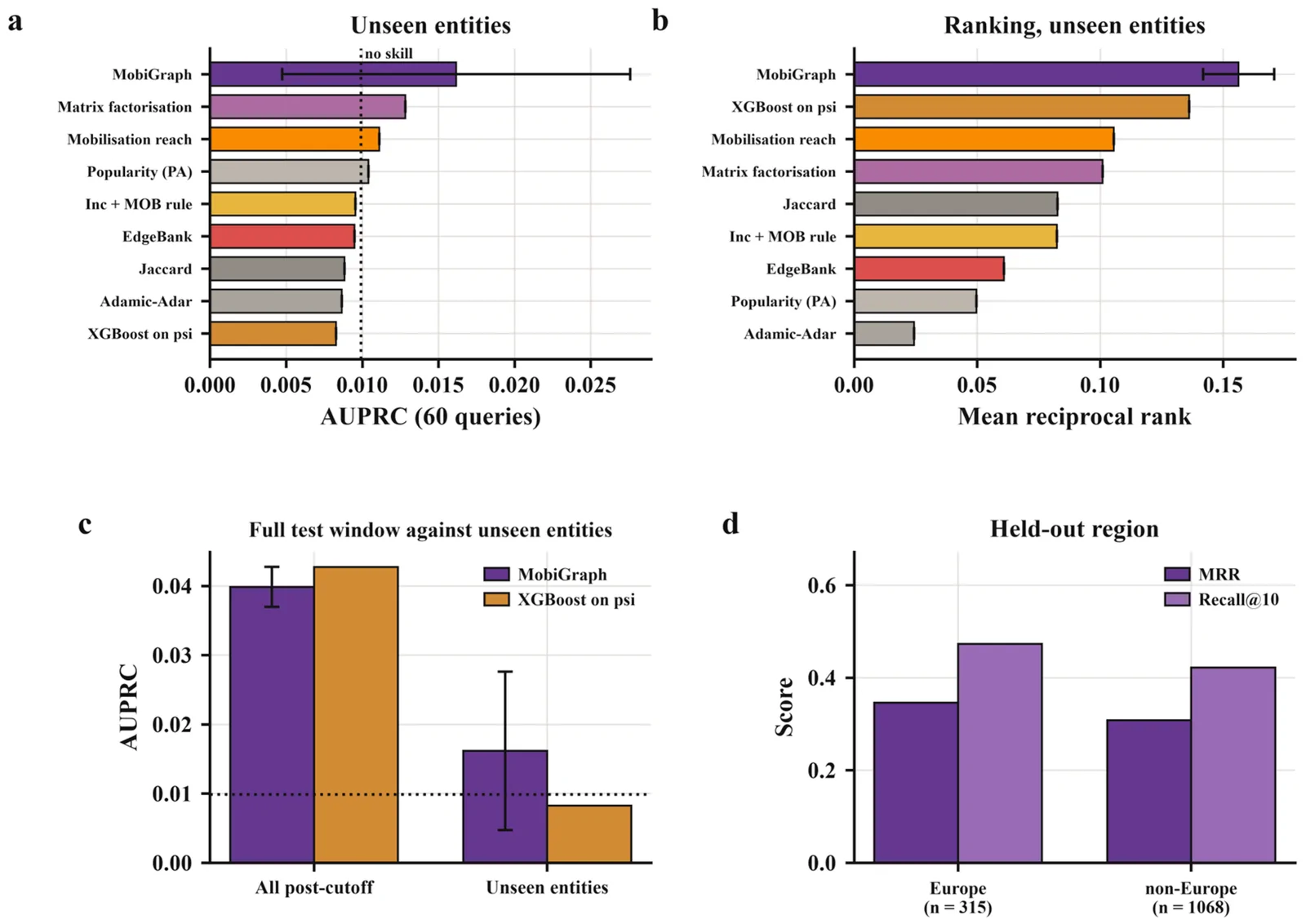
Generalisation to unseen entities and unseen regions. (**a**) AUPRC on the 60 inductive queries, in which at least one entity was unseen before the cutoff; the dotted line marks the no-skill level. (**b**) Mean reciprocal rank on the same queries. (**c**) AUPRC of MobiGraph and gradient boosting on the full post-cutoff test window and on the unseen-entity subset. (**d**) Mean reciprocal rank (MRR) and recall within the top ten (Recall@10) for European (n = 315) and non-European (n = 1068) triples under the geographic holdout.

The stratum that matters most for anticipating genuinely new events is the one in which at least one of the three entities was absent from the training window. On those 60 queries MobiGraph reached a mean reciprocal rank of 0.1563 with a standard deviation of 0.0144, ahead of gradient boosting at 0.1363 and of every other method, with matrix factorisation next at 0.1010 and the deposition-volume baseline at 0.0498 (Table 4, Figure 4b). The lead on reciprocal rank held at all three seeds. With 60 queries the interval around that figure is wide, 0.089 to 0.266 at the best seed, and it overlaps the interval for gradient boosting, 0.066 to 0.221 (Figure S13b). This stratum is therefore consistent with the pooled result rather than independent evidence of an advantage over it, and the comparison should be read as supportive. Area under the precision–recall curve averaged 0.0162 against 0.0083 for gradient boosting (Figure 4a), and recall within the top ten reached 17.2%. Moving from the full test window to this subset separates the two approaches: gradient boosting depends on the accumulated history of an entity, and where a gene, backbone or host has no history there is nothing for its mechanistic features to summarise, whereas the graph continues to propagate information from neighbouring nodes (Figure 4c). Generalisation across geography was assessed by assigning every triple to the region in which it was first observed. Evaluated on 315 European and 1068 non-European triples, reciprocal rank was 0.3463 and 0.3082, both close to the pooled figure, with recall within the top ten at 47.3% and 42.2% (Figure 4d and Figure S14, Table 4). A model exploiting the geography of sequencing rather than the biology of transfer would not hold its position on a region held out of training, and the two figures sit within 0.04 of each other.

### 3.5. Attaching an Error Rate That Survives the Temporal Boundary

**Figure 5:**
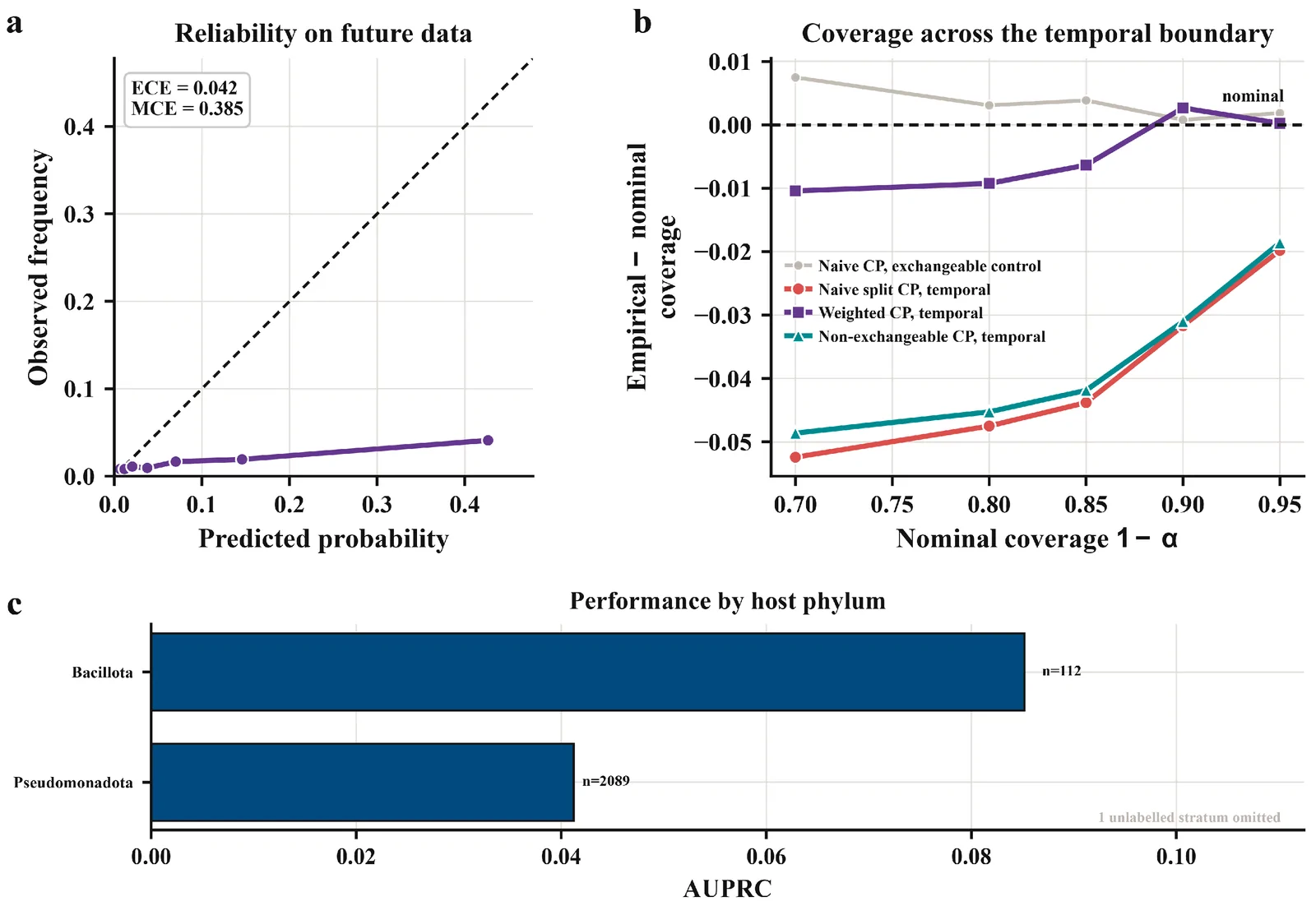
Calibration and distribution-free risk control across the temporal boundary. (**a**) Reliability diagram of predicted probability against observed frequency on the test window; the dashed line marks perfect calibration (ECE, expected calibration error; MCE, maximum calibration error). (**b**) Difference between empirical and nominal coverage for conformal prediction (CP) variants across nominal coverage levels; the exchangeable control calibrates and evaluates within the post-cutoff window, whereas the temporal arms calibrate on the validation year and evaluate on the test years. (**c**) AUPRC by host phylum, with stratum sizes (n) shown beside the bars.

A forecast handed to a laboratory with a finite follow-up budget needs a stated error rate. The raw model tracks the diagonal closely through the low-probability bins that hold most of the mass, moving from a predicted 0.0012 against an observed 0.0054 to a predicted 0.038 against an observed 0.0095 in the highest bin (Figure 5a). Separating the effect of the temporal boundary from everything else requires two calibration arms. Calibrated and evaluated on random halves of the same post-cutoff period, naive split conformal held its nominal level exactly, reaching 0.9008 against a nominal 0.90 (Table 5). Calibrated on the validation year and evaluated on the later test years, which is the situation the deployed system is in, reweighting the calibration scores by an estimated likelihood ratio between the two eras returned coverage to 0.9027 against the same nominal level, which held at every level tested, reaching 0.9502 against a nominal 0.95 and 0.7908 against 0.80 (Figure 5b, Table 5). The correction is specific to the covariate shift a temporal split creates, and identifying which of the two shifts operates here is what the paired arms establish. Because a guarantee met on average can conceal uneven performance, calibration was repeated independently inside each cell of a taxonomy defined by host phylum, drug class and predicted mobility. Among strata with enough positive cases to evaluate, area under the precision–recall curve reached 0.0852 in *Bacillota* across 14,163 candidates and 0.0412 in *Pseudomonadota* across 206,924, with expected calibration error of 0.0362 and 0.0437 respectively (Figure 5c, Table S10). The stratified procedure returns a shortlist inside every cell rather than covering the best-sampled ones (Figure S15a), and the twelve best-controlled strata span both phyla and several drug classes (Figure S15b). Applied to the whole candidate set, shortlist width trades directly against yield: the top 0.5% of candidates carries a 12.7-fold enrichment over the base rate, the top 1% a 9.4-fold enrichment at 9% recall, and the top 3% a 5.7-fold enrichment (Figure S16a). Shortlist size grows from 910 candidates at the strictest attainable level to 3293 and then 7980 as the level is relaxed, with the shift-corrected stratified variant returning the smallest set at matched yield (Figure S16b,c, Table S11). Ranked by score alone, flagging the top 0.1% gives 12.7-fold enrichment at 12.6% precision and 6.4% recall, which is the operating point a triage programme would adopt.

### 3.6. Reading Mobilisation Rules out of the Scoring Head

**Figure 6:**
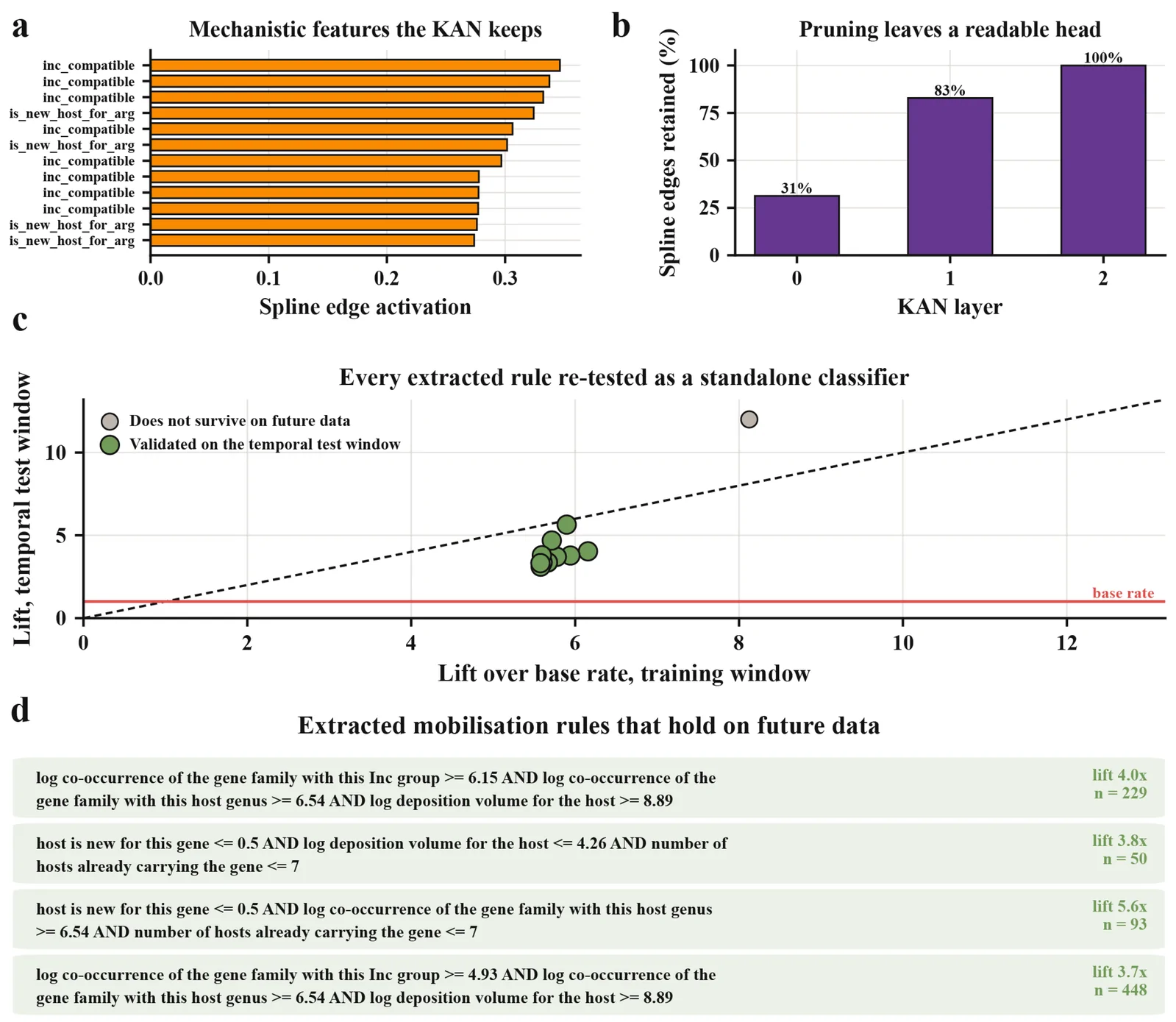
Rules extracted from the pruned scoring head and re-tested on future data. (**a**) Spline connections with the highest mean activation in the Kolmogorov–Arnold network (KAN) scoring head, labelled by mechanistic input feature. (**b**) Percentage of spline connections retained in each KAN layer after pruning. (**c**) Lift of each extracted rule over the base rate in the training window against its lift on the temporal test window; the dashed line marks equal lift and the red line the base rate. (**d**) The four best-supported rules that hold on future data, with their lift and support (n) on the test window.

Because the spline head costs nothing in accuracy, it can be pruned and read. Removing connections whose mean absolute activation fell below five percent of the largest in their layer eliminated 69% of the spline connections, retaining 31% at the input layer, 83% at the middle layer and all of them at the output, so the compression acts as feature selection rather than as a general thinning of the network (Figure 6b). Among the named mechanistic quantities, the surviving connections are led by incompatibility-group compatibility at a summed importance of 6.58 and by whether the host is new for the gene at 5.64, followed by whether the host already carries the same relaxase class at 2.23, the gene family’s history on that backbone class at 1.65, the guanine-cytosine differential at 1.56, the family’s history in that host genus at 1.51, and distance to the nearest origin of transfer and host deposition volume at 1.30 each (Figure 6a). Deposition volume ranks last among the retained features, which is the ordering the matched comparison was built to make possible. Reading the head as rules turns those weights into statements that can be checked. Enumerating 5276 conjunctive threshold rules over the retained features at data quantiles, keeping the twelve with the highest enrichment on the training window, and rescoring each as a standalone classifier on the temporal test window left eleven with enough support to act on (Figure 6c); the full set, with support, precision and lift on both windows, is given as Table S12. The strongest combines history with sampling depth—where a gene family has been seen often on a given incompatibility group, often in a given host genus, and the host is well-sequenced, enrichment on future data is 4.03-fold across 229 candidates. A second reads mechanistically: for a gene already present in a host but on few other hosts, and with strong family-level history in that genus, enrichment reaches 5.6-fold across 93 candidates. Thresholds for the four best-supported rules are given in their original units of kilobases, patristic distance and log counts, so each is a statement a microbiologist can evaluate independently of the model that produced it (Figure 6d).

### 3.7. Anticipating Events the Field Already Knows About, and How Far Ahead

**Figure 7:**
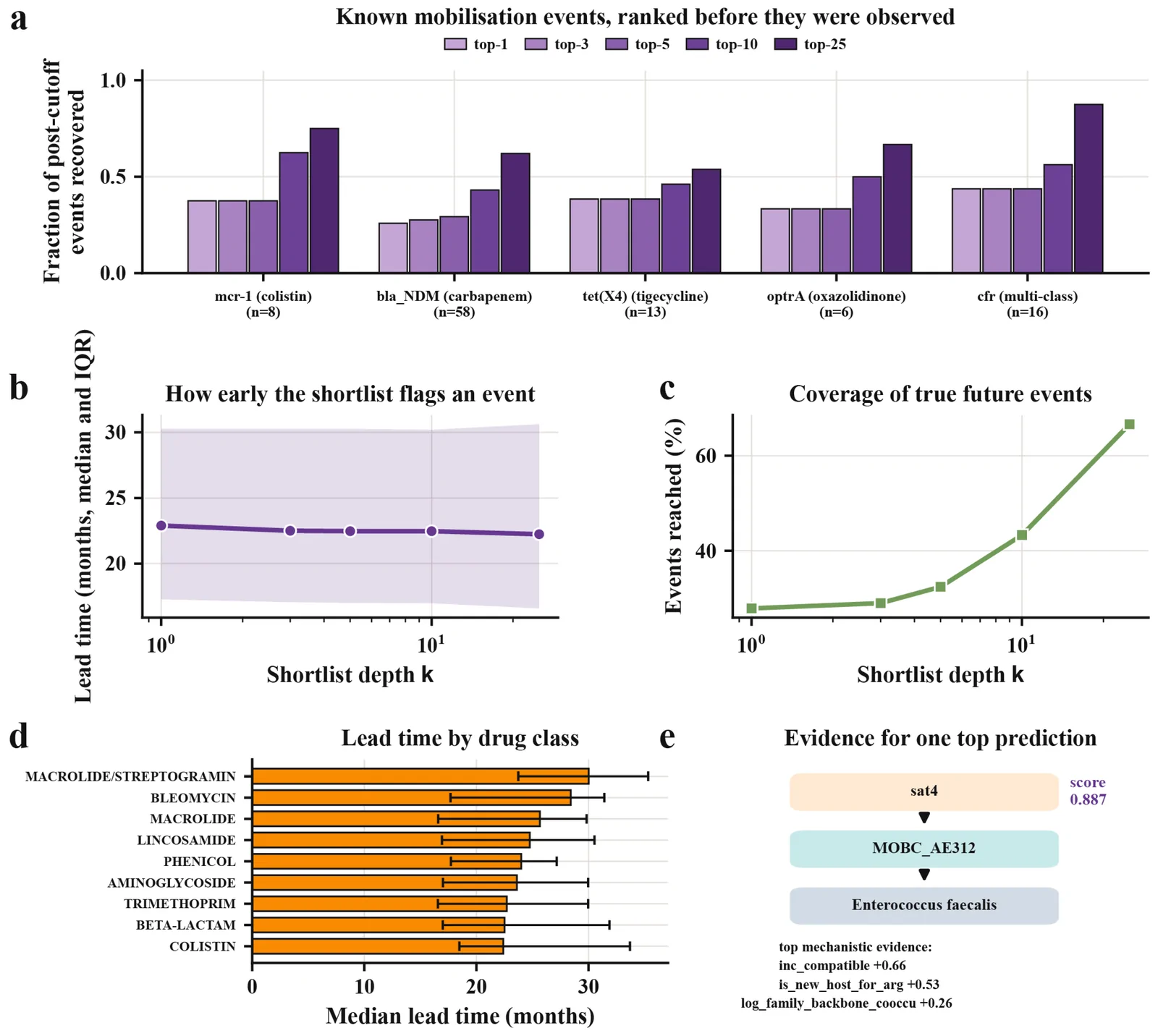
Known mobilisation events and lead time. (**a**) Fraction of post-cutoff events recovered within the top 1, 3, 5, 10 and 25 candidates for five documented resistance determinants; n is the number of post-cutoff events. (**b**) Lead time (median and interquartile range, in months) of recovered events against shortlist depth k. (**c**) Percentage of true future events reached at each shortlist depth. (**d**) Median lead time by drug class; error bars show the interquartile range. (**e**) Mechanistic evidence for one top-scoring prediction, *sat4* on the MOBC_AE312 backbone in *E. faecalis*.

Five mobilisations that the field has documented provide an external check: mcr-1 on IncI2 and IncHI2 backbones, blaNDM on IncX3, tet(X4), optrA on enterococcal plasmids, and cfr. Each post-cutoff event was scored inside a pool holding one true host and 100 effort-matched alternatives. The model placed 62% of post-cutoff mcr-1 events in the top ten of their pools at a median rank of 6, 56% of cfr events at median rank 8, 50% of optrA at median rank 12, 46% of tet(X4) at median rank 24 and 43% of blaNDM at median rank 12 (Figure 7a, Table 6). The recovery curves separate early and remain ordered across depths, so the result does not depend on the depth chosen. Between 6 and 58 post-cutoff events are available per determinant, and the intervals are correspondingly wide: 62% (95% CI 31 to 86) for mcr-1, 56% (34 to 77) for cfr, 50% (19 to 81) for optrA, 46% (23 to 71) for tet(X4) and 43% (31 to 56) for blaNDM (Table 6). These five determinants test whether the model behaves sensibly on mobilisations the field has already characterised; they are not an independent estimate of its accuracy.

The quantity a surveillance programme acts on is lead time. Among true post-cutoff triples entering the top ten of their pool at the cutoff, the median interval to first observation was 22.5 months, with an interquartile range of 17.0 to 30.2 months, and every recovered event carried a positive lead time (Figure 7b). At that depth 43.3% of true events were reached (Figure 7c). Lead time was stable across drug classes with enough events to estimate it, running from 19.1 months for aminoglycoside-quinolone determinants and 19.7 for tetracyclines through 22.5 for beta-lactams and 23.6 for aminoglycosides to 28.4 for bleomycin and 30.0 for macrolide-streptogramin determinants (Figure 7d, Table S13). Consistency across mechanistically unrelated classes indicates the interval reflects the deposition-to-observation pipeline rather than a property of any one resistance mechanism. Individual predictions can be read the same way as the rules: for sat4 on a MOBC backbone in *E. faecalis*, scored at 0.887, incompatibility compatibility contributed 0.66, the host being new for that gene 0.53, the gene family’s history on that backbone class 0.26 and the relaxase class already present in the host 0.22 (Figure 7e).

### 3.8. A Frozen Watch List, and What Happened to It

**Figure 8:**
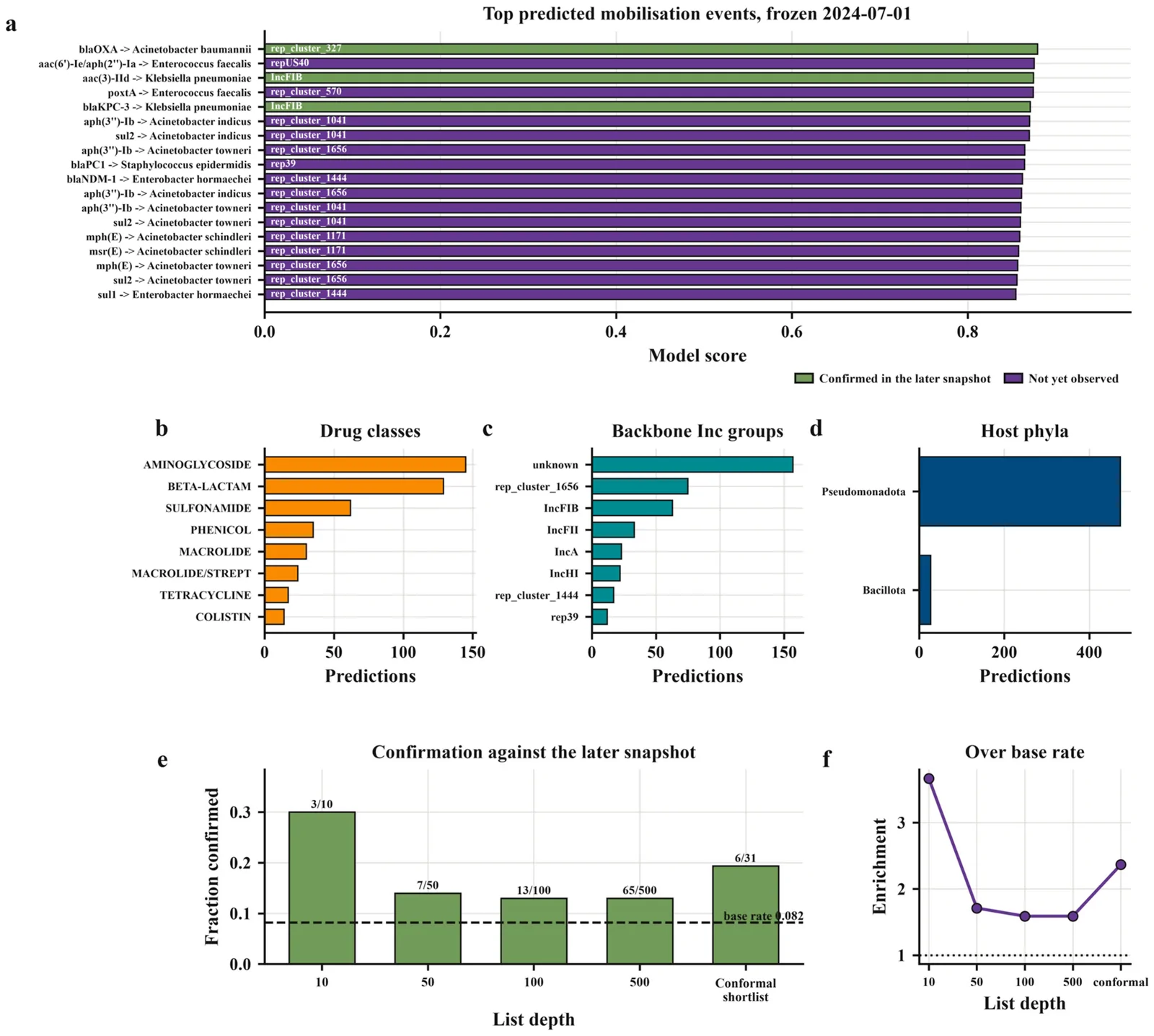
The frozen watch list and its confirmation. (**a**) Highest-scoring entries of the watch list frozen on 1 July 2024, labelled by gene and host, with the predicted backbone shown inside each bar; green bars mark pairs confirmed in the later snapshot. (**b**) Drug classes of the 500 entries. (**c**) Backbone incompatibility groups of the 500 entries. (**d**) Host phyla of the 500 entries. (**e**) Fraction of entries confirmed in the later snapshot at increasing list depths and for the conformal shortlist; the dashed line marks the 8.2% base rate. (**f**) Enrichment over the base rate at each list depth.

The model was frozen at the corpus cutoff of 1 July 2024 and used to score 332,795 unobserved triples restricted to phylogenetically plausible and adequately sequenced hosts. The 500 highest-scoring were written with a cryptographic digest and a freeze manifest recording the model, the cutoff and the time of freezing. The highest-scoring entries are dominated by aminoglycoside and beta-lactam determinants moving among *Acinetobacter* and *Enterococcus* species on backbones already carrying related genes (Figure 8a). Across the list, aminoglycoside determinants account for 145 entries and beta-lactams 129, followed by sulfonamides at 62, phenicols at 35 and macrolides at 30 (Figure 8b); the largest identifiable backbone families are IncFIB with 63 entries and IncFII with 33 (Figure 8c); and hosts are predominantly *Pseudomonadota* at 473 entries against 27 *Bacillota* (Figure 8d), mirroring the composition of the corpus. Several entries are biologically unsurprising, which is what a screening instrument should produce: blaKPC-3 on an IncFIB backbone in *Klebsiella pneumoniae*, and poxtA on an enterococcal backbone in *E. faecalis*. Scoring that unchanged file against a later surveillance snapshot, after verifying its digest, gives the study its external test. Over the 25 months between the corpus cutoff and the snapshot of 7 August 2026, 65 of the 500 predictions were confirmed by the appearance of the predicted gene in the predicted host, 13.0% (95% CI 10.3 to 16.2) against a base rate of 8.18% obtained by applying the identical criterion to all 332,795 scored candidates, a difference that would arise by chance with probability 1.6 × 10^−4^ (one-sided binomial test; Table 7, Figure 8e). Pathogen Detection does not resolve the replicon, so these are gene–host confirmations, and the predicted backbone remains untested. Confirmation concentrates at the top of the list, where 3 of the first 10 and 13 of the first 100 were observed, giving 3.7-fold and 1.6-fold enrichment over the base rate, and the conformal shortlist returned 6 confirmations among 31 candidates, a 2.4-fold enrichment (Figure 8f). At these depths the counts are small and only some of the enrichments separate from the background on their own: *p* = 0.042 at depth 10, *p* = 0.064 at depth 100 and *p* = 0.037 for the conformal shortlist (Table S15). Three of the five highest-scoring predictions were among those confirmed: blaOXA in *Acinetobacter baumannii* at rank one, aac(3)-IId in *K. pneumoniae* at rank three, and blaKPC-3 in *K. pneumoniae* at rank five.

## 4. Discussion

Posed as a dated link prediction problem, the first record of a resistance gene in a new host turns out to be anticipable at a horizon that is useful to a surveillance programme. Trained on nothing later than the cutoff and scored against alternatives matched for how heavily they have been sequenced, the model placed the correct host within the top ten of a hundred candidates in 42% of cases, a median of roughly two years before the combination was first recorded anywhere. The five mobilisations the field already knows about behave the same way, with between 43% and 62% of their post-cutoff appearances recovered at that depth. A frozen list of 500 unobserved combinations, digested and manifested before any later snapshot was consulted, has since accumulated 65 gene–host confirmations against a background rate of 8%, with three of its first ten entries observed. Taken together, these indicate that the question a surveillance officer asks, which organism to examine next, admits a quantitative answer rather than only a retrospective one. That answer concerns the next database record. Whether a combination appearing for the first time has just been acquired, or has been present in that host and not yet sequenced at plasmid resolution, is not something these data can decide, and the wording throughout has been chosen to respect the distinction. What the model relies on to produce that answer is recognisably the biology. Incompatibility-group compatibility is the strongest quantity surviving the pruning of the scoring head, which follows from replicon incompatibility acting as a genuine barrier to stable coexistence [73,74]. Relaxase class and the presence of an origin of transfer come next, consistent with mobilisation being gated by conjugative machinery that may be supplied in trans [9,11,42,43]. Deposition volume ranks last among the retained quantities. That ordering is an internal check worth more than any single accuracy figure, since it indicates the model is using transfer mechanisms rather than sampling intensity, and it is why the rules read off the head were re-tested as standalone classifiers on future data rather than simply displayed [69,70]. Eleven of twelve survived, giving a compact set of rules expressed in kilobases and patristic units that a microbiologist can evaluate without reference to the model that produced it.

The graph contributes most where the mechanistic picture predicts it should. On entities absent from the training window, a tree over per-candidate features has little to summarise, while a graph model can still place a new entity by its neighbours, and the ranking advantage on that subset held at every seed, though with 60 queries the intervals for the two methods overlap and the stratum is offered as supporting evidence rather than as an independent result. Those cold-start cases are the ones a surveillance programme most needs to catch, since a genuinely new gene, backbone or host is where an unanticipated association would first appear, and reporting them as a separate stratum is what keeps that capability visible instead of diluting it into a pooled average. For a group with no appetite for temporal graph infrastructure, a practical reading is that five interpretable quantities, namely relaxase class, origin of transfer, incompatibility compatibility, distance to the nearest mobility marker and patristic distance to the current host range, fed to a gradient-boosted tree will reach much of this performance, and graph context can be added where entities are new. Attaching a defensible error rate required distinguishing two things that are easily confused. Split conformal prediction held its nominal level when calibration and evaluation were drawn from the same period, and reweighting the calibration scores by an estimated covariate likelihood ratio held it across a genuine past-to-future boundary, returning 0.903 against a nominal 0.90 and tracking the nominal level at every other tested value. Weights that decay with the age of the calibration point behaved differently, which localises what moves in this corpus: the distribution of candidates being proposed, rather than the rate at which recent observations become stale. Only a paired comparison of the two arms makes that distinction visible, and it seems likely to recur in any forecast built on an archive that grows unevenly.

Ellabaan and colleagues identified mobilisation elements statistically and predicted how far known resistance genes could reach phylogenetically, confirming a subset in an independent genome collection [22]; their target is clade reach at coarse taxonomic resolution and their confirmation set is a different database, whereas the target here is a named gene on a named backbone in a named host, confirmed against a later time window. Function-based approaches predict which bacterial pairs have exchanged genes from their functional content and report near-perfect discrimination [23] on a protocol that censors edges at random from a fixed network and predicts a pair of organisms rather than a triple. A separate and now dependable literature predicts resistance phenotype from a finished genome [24,25,26], answering what an organism can already resist rather than what it is about to acquire. The results here indicate that figures in this area are comparable within a protocol, and that stating the protocol alongside the number is what makes cross-study comparison possible [27,28,30,31,75].

Several extensions follow from the design and are supported by the released pipeline. De novo annotation of insertion sequences and integrons across the assemblies would enrich the context block, particularly for the distance-to-nearest-element quantities the symbolic head made most use of [11,76,77]. Host defence repertoire is the natural next feature block, since restriction-modification and CRISPR systems plausibly determine whether a transferred replicon is retained, and both are annotatable at scale [78,79]. Conjugation assays on a handful of top-ranked triples would carry a computational shortlist to the bench, and the quantities the model relies on are precisely those such an assay probes. The watch list itself remains the most direct test available: frozen with a digest and a manifest, it can be rescored against each new database release without changing anything else in the analysis, and the confirmations already recorded will either accumulate or they will not.

### Limitations

What is predicted here is the first appearance of a gene–backbone–host combination in a public database. A newly recorded association may follow recent acquisition, or it may be a long-standing one that nobody has yet sequenced at plasmid resolution, and nothing in these data separates the two; lead time is accordingly a lead over the record rather than over the biology. Candidate hosts never seen with a gene are treated as negatives, but an unobserved combination may be a true absence, an association not yet observed, or an artefact of where sequencing has been done: a third of the sampled candidates are pairs the surveillance layer has already recorded without a resolved plasmid. The task is therefore closer to positive–unlabelled learning than to binary classification. Area under the precision–recall curve, precision and the false-discovery proportion the conformal layer controls are conservative, the risk guarantee holds for the labels as defined here rather than for biological absence, and the ranking metrics are displaced in the same direction by an amount these data cannot quantify. The corpus reflects deposition practice as much as biology. Three countries supply 60% of located records, the Gini coefficient of records per backbone is 0.81 and of isolates per host taxon 0.79, and the surveillance layer is built from eight organism groups, so taxa outside them—environmental and commensal species in particular—are under-represented, whatever their part in dissemination. The replicon layer compounds this, since closed plasmid sequences come disproportionately from clinical isolates, reference collections and long-read projects and under-represent elements that are difficult to close. Effort-matched sampling removes the first-order advantage a model gains from favouring well-sequenced taxa, and the geographic holdout shows the forecast does not collapse outside Europe, but neither can recover a host that has not been sequenced. Both inputs to the graph are derived rather than given. Collection dates were reconstructed, with 71.8% of them from a primary source and 28.6% resolved to the day; Section 3.3 shows that the ranking result survives stratification by date route, restriction to better-resolved records and resampling of every imprecise date, but lead time inherits that resolution and is accurate to within a few months rather than to the week. Gene identity rests on a single caller, and although the forecast does not differ between genes the two catalogues share and genes they do not, an allele that AMRFinderPlus assigns incorrectly is assigned incorrectly throughout. Several strata are small—60 inductive queries, between 6 and 58 post-cutoff events per documented mobilisation, and 10 to 100 entries at the top of the watch list—and their intervals are correspondingly wide, so they support the pooled result rather than standing alone. The watch-list confirmation is limited in two further ways: it is at gene–host resolution, because Pathogen Detection does not resolve the replicon, so the backbone of each predicted triple remains untested, and it is a blinded out-of-time validation rather than a live prospective trial, since the archive scored against already existed when the list was fixed and digested. Rescoring the same file against future releases is prospective in the strict sense, and that is the test we propose.

## 5. Conclusions

The host in which a resistance gene is next recorded, posed as a dated link prediction problem over alleles, backbones and host taxa, is predictable at a horizon a surveillance programme can act on. Trained only on what was known before a fixed cutoff and scored against alternatives matched for sequencing effort, MobiGraph placed the correct host within the top ten of a hundred candidates in 42% of post-cutoff cases, a median of 22.5 months before the combination was first recorded, and recovered between 43% and 62% of the post-cutoff appearances of five mobilisations the field has already documented. The advantage over feature-based methods is an advantage in ranking, and it is largest on entities with no prior history, which is the situation an unanticipated association presents, although that stratum is small enough that it supports the pooled result rather than standing on its own. The forecast comes with the two things that make it usable. Reweighting the conformal calibration for the covariate shift a temporal split creates returns coverage to its nominal level across the boundary between the data the model learned from and the data it is applied to. Pruning the spline scoring head yields eleven threshold rules that survive re-testing on future data, stated in kilobases and patristic units and led by incompatibility-group compatibility rather than by sequencing volume, so a prediction can be inspected rather than only trusted. A frozen list of 500 unobserved gene, backbone and host combinations, released with a cryptographic digest and a freeze manifest, has accumulated 65 gene–host confirmations against an 8% background rate, three of them among its ten highest-scoring entries. What such a list anticipates is the next database record, which is the quantity a surveillance programme can act on and is not the same as the transfer event behind it. That list can be rescored against every future database release, and it is the test on which this approach should ultimately be judged.

## Figures and Tables

**Table 1:** Composition of the tripartite mobilisation graph and of the surveillance layer used to estimate deposition effort.

Quantity	Value
Plasmid records (PLSDB 2024_05_31_v2)	72,556
Records carrying at least one acquired ARG	20,419
ARG observations (record × allele)	99,910
ARG allele nodes	458
Replicon backbone nodes	574
Host species nodes	222
Mobile-element family nodes	285
Timestamped edges	731,686
Records with a recovered collection date	71.8%
Collection dates at day resolution	28.6%
Collection date range	1 July 1951 to 1 July 2024
Countries represented	126
Host species on the GTDB reference tree	178/222
Surveillance isolates (NCBI Pathogen Detection)	2,116,241
Isolates with a collection date	85.6%
Species-intrinsic alleles excluded	20
Surveillance collection date range	1 January 1950 to 28 July 2026
Databases accessed	7 August 2026

**Table 2:** Prospective forecasting performance on the temporal test window under effort-matched negatives, over 2443 queries. MobiGraph is the mean and standard deviation across three seeds. Ranking metrics are primary; recall at depth 100 is near-saturated for every method at this pool size and is reported in Table S6. Bootstrap 95% confidence intervals for every entry are given in Table S7.

Method	AUPRC	AUROC	MRR	Recall@1	Recall@5	Recall@10
Popularity (PA)	0.0195	0.6462	0.2388	0.1907	0.2292	0.3385
Adamic-Adar	0.0102	0.5123	0.0323	0.0078	0.0082	0.0156
Jaccard	0.0091	0.4807	0.0540	0.0336	0.0413	0.0557
Inc + MOB rule	0.0158	0.6648	0.0936	0.0503	0.0774	0.1343
EdgeBank	0.0125	0.5977	0.0497	0.0143	0.0282	0.0679
Mobilisation reach	0.0130	0.6005	0.0601	0.0225	0.0385	0.0839
Matrix factorisation	0.0132	0.6153	0.1529	0.1154	0.1371	0.1985
XGBoost on psi	0.0428	0.7336	0.3071	0.2607	0.3000	0.4147
MobiGraph	0.0399 ± 0.0029	0.7122 ± 0.0043	0.3204 ± 0.0053	0.2744	0.3152	0.4242

**Table 3:** Ablation arms, each trained and evaluated under the identical protocol. The full model was run at three seeds and each arm at one.

Arm	Seeds	AUPRC	MRR	Recall@10	MRR Relative to Full
Full model	3	0.0399	0.3204 ± 0.0053	0.4242	reference
Node features reduced to a type indicator	1	0.0288	0.2741	0.3917	−14.4%
Without the mechanistic feature block	1	0.0490	0.2980	0.4032	−7.0%
Without host phylogeny features	1	0.0449	0.3054	0.4224	−4.7%
Without the time encoding	1	0.0332	0.3077	0.4220	−4.0%
One weight set shared across relations	1	0.0337	0.3247	0.4290	+1.3%
Without the latent-mechanism mixture	1	0.0357	0.3210	0.4400	+0.2%
Multilayer perceptron in place of the spline head	1	0.0350	0.3214	0.4421	+0.3%

**Table 4:** Generalisation to entities and regions held out of training. The upper block is the inductive subset, 60 queries in which at least one endpoint was unseen before the cutoff; the lower block evaluates triples first observed inside and outside Europe separately. The inductive block rests on 60 queries and its bootstrap intervals overlap those of the strongest baseline.

Method	AUPRC	MRR	Recall@10
Popularity (PA)	0.0104	0.0498	0.0500
Adamic-Adar	0.0087	0.0244	0.0667
Jaccard	0.0088	0.0827	0.0833
Inc + MOB rule	0.0095	0.0825	0.0667
EdgeBank	0.0095	0.0609	0.1000
Mobilisation reach	0.0111	0.1056	0.1500
Matrix factorisation	0.0128	0.1010	0.1000
XGBoost on psi	0.0083	0.1363	0.1500
MobiGraph	0.0162 ± 0.0114	0.1563 ± 0.0144	0.1722
MobiGraph, Europe	0.1018	0.3463	0.4730
MobiGraph, non-Europe	0.0365	0.3082	0.4223

**Table 5:** Empirical coverage against the nominal level. The exchangeable arm calibrates and evaluates on random halves of the post-cutoff window; the temporal arm calibrates on the validation year and evaluates on the later test years.

Nominal Coverage	Calibration Arm	Naive Split CP	Weighted CP	Non-Exchangeable CP
0.7000	exchangeable	0.7075	0.6830	0.7060
0.7000	temporal	0.6476	0.6896	0.6514
0.8000	exchangeable	0.8031	0.7837	0.8022
0.8000	temporal	0.7525	0.7908	0.7547
0.8500	exchangeable	0.8539	0.8387	0.8510
0.8500	temporal	0.8062	0.8437	0.8081
0.9000	exchangeable	0.9008	0.8921	0.8982
0.9000	temporal	0.8683	0.9027	0.8690
0.9500	exchangeable	0.9519	0.9476	0.9498
0.9500	temporal	0.9302	0.9502	0.9314

**Table 6:** Known mobilisation events scored inside their own matched candidate pools, each containing one true host and 100 effort-matched alternatives. Event counts are small; Wilson 95% confidence intervals for every recovery fraction are given in Table S14.

Gene Family	Post-Cutoff Events	Alleles	Median Rank	Recovered in Top 1	Recovered in Top 3	Recovered in Top 5	Recovered in Top 10	Recovered in Top 25	Candidates per Pool
mcr-1 (colistin)	8	4	6	38%	38%	38%	62%	75%	101
bla_NDM (carbapenem)	58	5	12	26%	28%	29%	43%	62%	101
tet(X4) (tigecycline)	13	1	24	38%	38%	38%	46%	54%	101
optrA (oxazolidinone)	6	1	12	33%	33%	33%	50%	67%	101
cfr (multi-class)	16	2	8	44%	44%	44%	56%	88%	101

**Table 7:** Confirmation of the frozen watch list against a later surveillance snapshot. A candidate counts as confirmed when its gene and host were observed together in an isolate collected after the corpus cutoff. The base rate applies the identical criterion to all 332,795 scored candidates. Confirmation is at gene–host resolution, since Pathogen Detection does not resolve the replicon. Intervals are Wilson intervals and *p* values are one-sided binomial tests against the base rate.

List Depth	Candidates	Confirmed	Fraction Confirmed (95% CI)	Base Rate	Enrichment (One-Sided *p*)
10	10	3	0.300 (0.108–0.603)	0.0820	3.67 (*p* = 0.042)
50	50	7	0.140 (0.070–0.262)	0.0820	1.71 (*p* = 0.111)
100	100	13	0.130 (0.078–0.210)	0.0820	1.59 (*p* = 0.064)
500	500	65	0.130 (0.103–0.162)	0.0820	1.59 (*p* = 1.6 × 10^−4^)
conformal shortlist	31	6	0.194 (0.092–0.363)	0.0820	2.37 (*p* = 0.037)

## Data Availability

The graph, the analysis code, the trained model, the extracted rules and the frozen watch list with its digest are available at https://github.com/waaljohani/MobiGraph. All primary data are public: plasmid records and their annotation from PLSDB release 2024_05_31_v2, isolate metadata from NCBI Pathogen Detection, resistance gene references from the NCBI AMRFinderPlus Reference Gene Catalog database version 2026-05-15.1 and the Comprehensive Antibiotic Resistance Database, and taxonomy and the bac120 reference tree from the Genome Taxonomy Database release 232. Every source was accessed on 7 August 2026, and the NCBI Pathogen Detection snapshots are PDG000000004.6221 for *E. coli*/*Shigella*, PDG000000002.4177 for *Salmonella*, PDG000000012.2484 for *Klebsiella*, PDG000000010.1917 for *Acinetobacter*, PDG000000071.928 for *E. faecium*, PDG000000073.1253 for *S. aureus*, PDG000000036.1750 for *P. aeruginosa* and PDG000000003.2870 for *Campylobacter*. Exact release identifiers for every source are recorded in a version manifest distributed with the code. An archived release of the repository, version 1.0.0, is deposited in Zenodo at https://doi.org/10.5281/zenodo.23101065.

## Supplementary Materials

The following supporting information can be downloaded at https://www.mdpi.com/article/10.3390/genes17101291/s1. Figure S1: The analysis pipeline in outline; Figure S2: provenance and resolution of the reconstructed collection dates; Figure S3: gene calling and the species-intrinsic filter; Figure S4: sampling concentration in the corpus; Figure S5: covariate balance of the effort-matched sampler; Figure S6: status of the sampled candidate hosts; Figure S7: performance under uniform and effort-matched negatives; Figure S8: ablation arms; Figure S9: training behaviour; Figure S10: computational cost; Figure S11: sensitivity to collection-date uncertainty; Figure S12: robustness to the choice of gene caller; Figure S13: uncertainty intervals on every method and stratum; Figure S14: geographic holdout; Figure S15: stratified risk control; Figure S16: shortlist operating characteristics; Table S1: a glossary of the modelling terms; Table S2: caller concordance; Table S3: the fifteen commonest resistance determinants in the corpus; Table S4: corpus composition by host phylum; Table S5: negative sampling regimes; Table S6: recall at the deeper positions of the candidate pool; Table S7: ranking metrics with 95% confidence intervals for every method; Table S8: forecast performance under alternative treatments of date uncertainty; Table S9: expanded caller concordance and the commonest unmatched calls; Table S10: performance by host phylum; Table S11: shortlist size and yield; Table S12: the full set of threshold rules read from the scoring head; Table S13: lead time by drug class; Table S14: known mobilisation events with confidence intervals; Table S15: watch-list confirmation with confidence intervals and tests.
