# Supplementary material for: Anticipating the Next Recorded Bacterial Hosts of Plasmid-Borne Antimicrobial Resistance Genes: A Temporal Graph Analysis of Plasmid and Surveillance Data
Source: Genes (Basel). 2026 Oct 15;17(10):1291. doi: 10.3390/genes17101291 (PMC13704231; doi:10.3390/genes17101291)
Supplement: Supplementary file 1 [file genes-17-01291-s001.zip › genes-4597799-supplementary.pdf]

Supplementary Materials

Anticipating the Next Recorded Bacterial Hosts of Plasmid-Borne Antimicrobial Resistance Genes: A Temporal Graph Analysis of Plasmid and Surveillance Data

Waad A. Aljohani

Department of Basic Health Sciences, College of Applied Medical Sciences, Qassim University, Buraydah 51452, Saudi Arabia; wa.aljohani@qu.edu.sa

Supplementary Figures

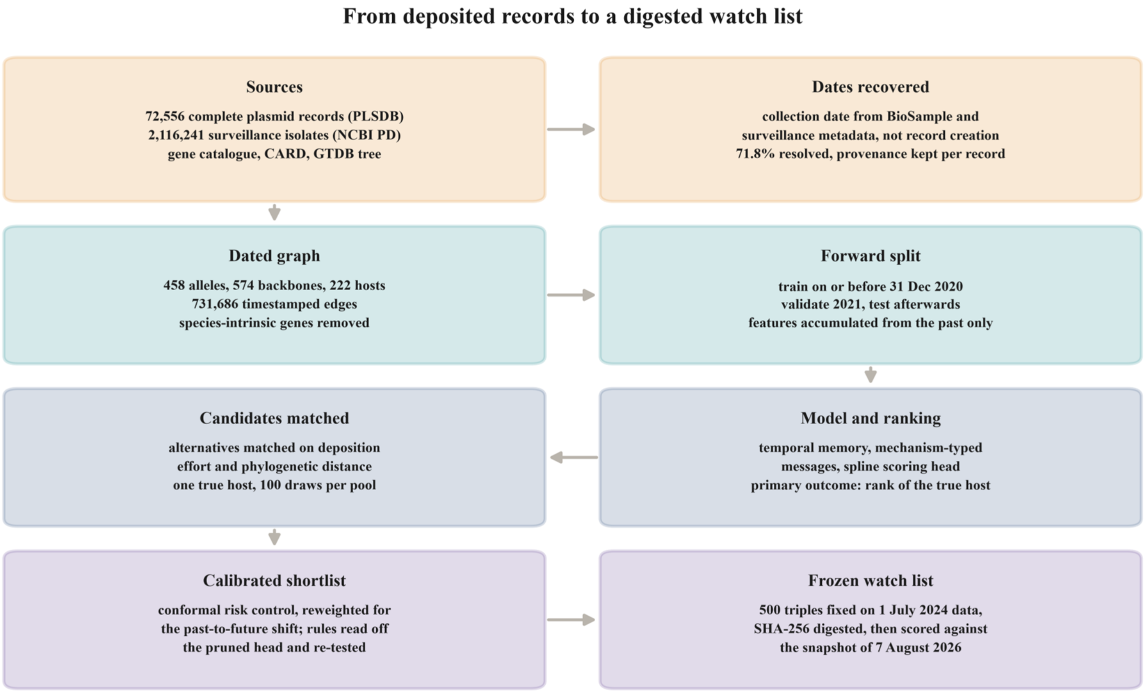

Figure S1. The analysis in outline, from the deposited records to the frozen watch list. Each box names the step and the quantities that define it; the arrows follow the order in which the numbered pipeline stages run.

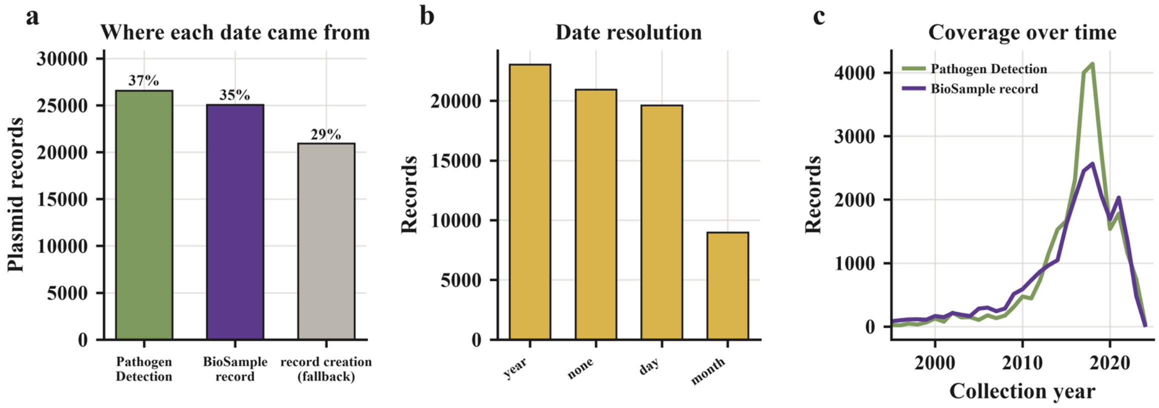

Figure S2. Provenance and resolution of the reconstructed collection dates. a Route by which each date was recovered. b Resolution of the recovered dates. c Coverage of dated records over time.

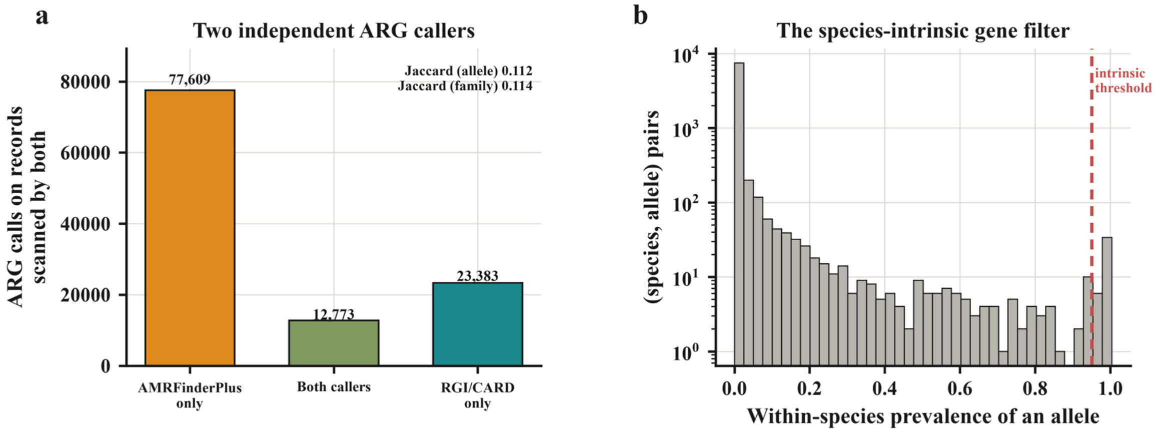

Figure S3. Gene calling and the species-intrinsic filter. a Agreement between the two resistance gene callers on records scanned by both. b Alleles removed by the species-intrinsic prevalence rule.

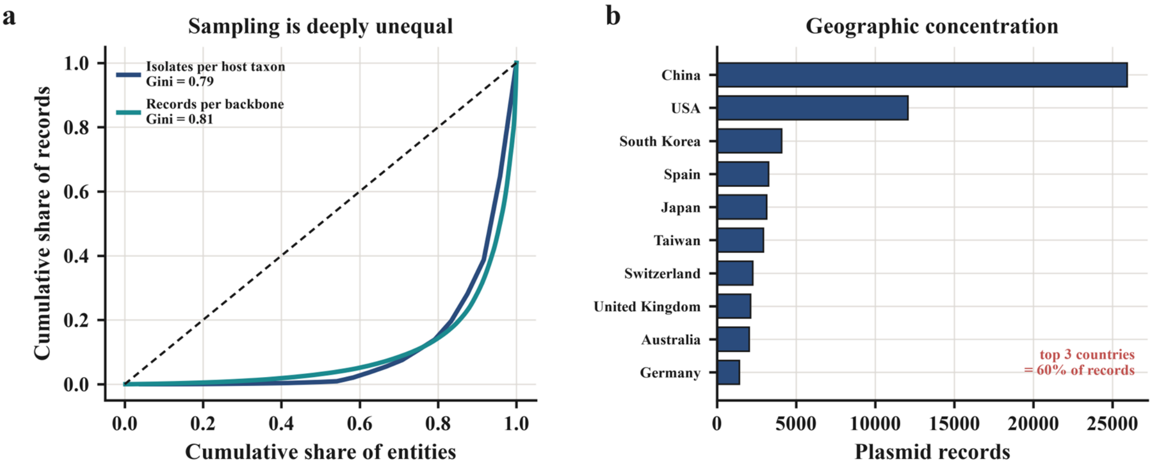

Figure S4. Sampling concentration in the corpus. a Lorenz curves for isolates per host taxon and records per backbone, with Gini coefficients. b Plasmid records by country, excluding records with no recorded location.

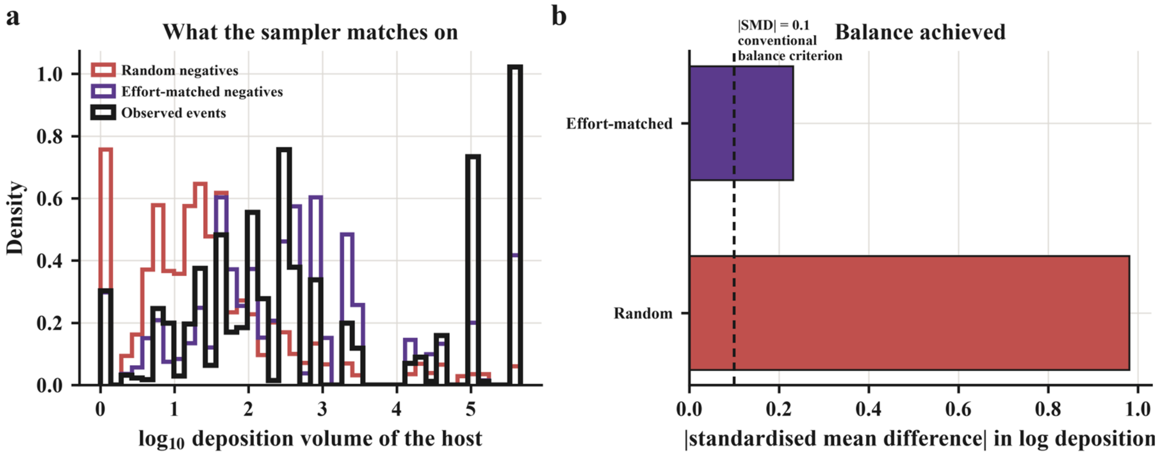

Figure S5. Covariate balance of the effort-matched negative sampler. a Quantities the sampler matches on. b Balance achieved between true and sampled hosts.

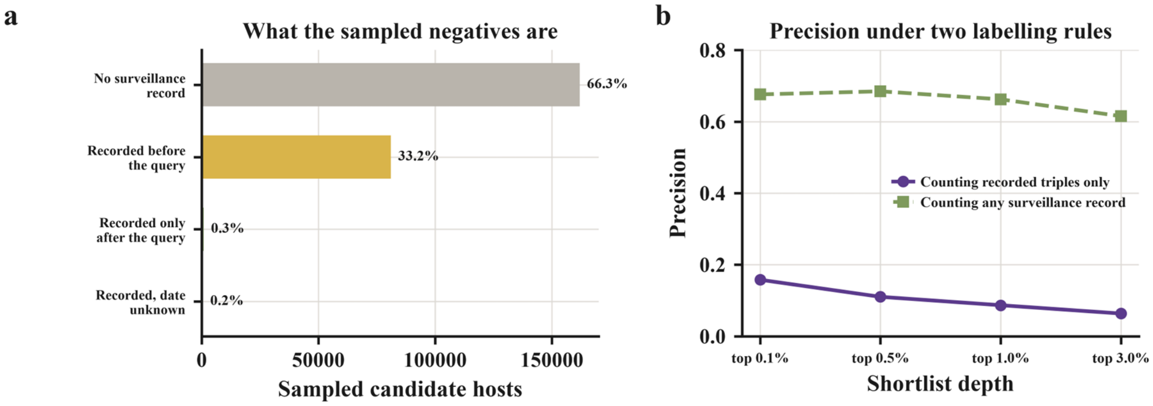

Figure S6. What the sampled candidate hosts are. a Candidates drawn as negatives for the test pools, classified by whether the surveillance layer records that gene–host pair and, if so, whether the record predates the query. b Precision of the ranked shortlist counting only recorded triples, and counting any surveillance record of the pair, which bounds the effect of unlabelled positives.

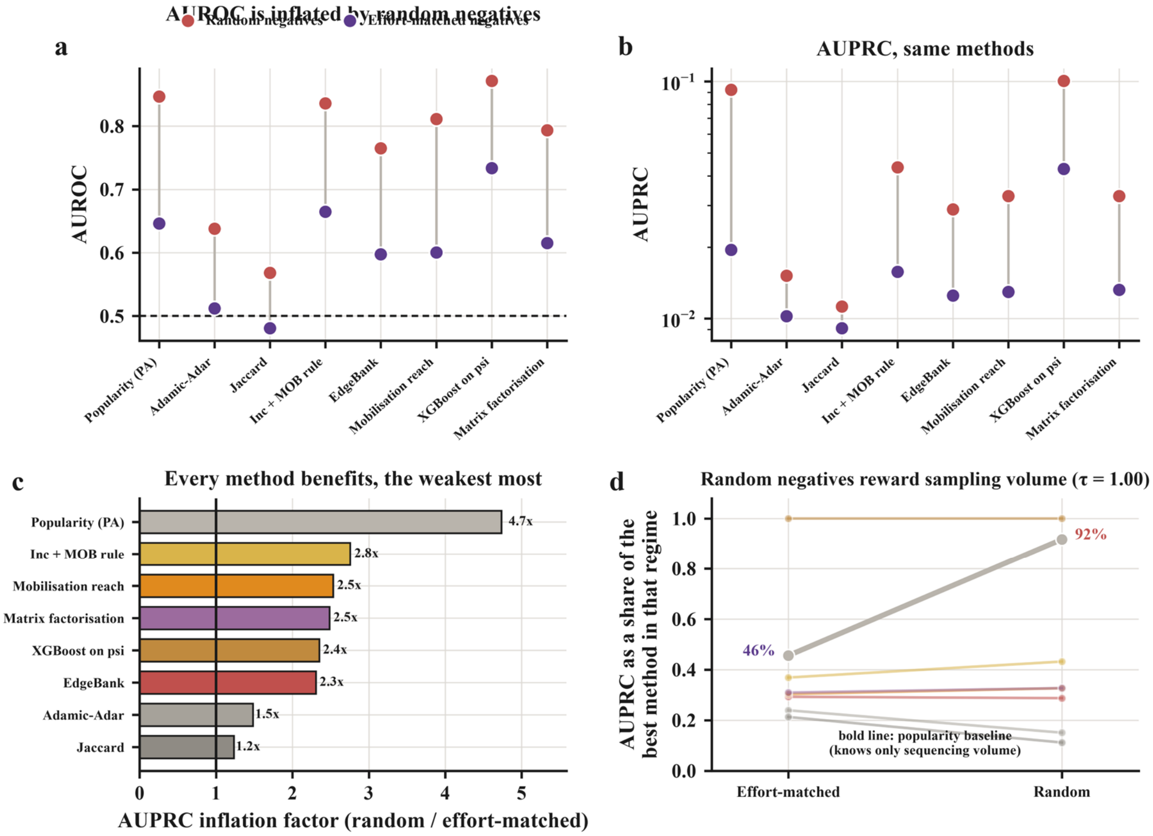

Figure S7. Performance under uniformly drawn against effort-matched negatives, for identical positive examples. a Area under the receiver operating characteristic curve; the dashed line is chance. b Area under the precision-recall curve, logarithmic scale. c The ratio of the two, by method. d Each method as a share of the best method in the same regime.

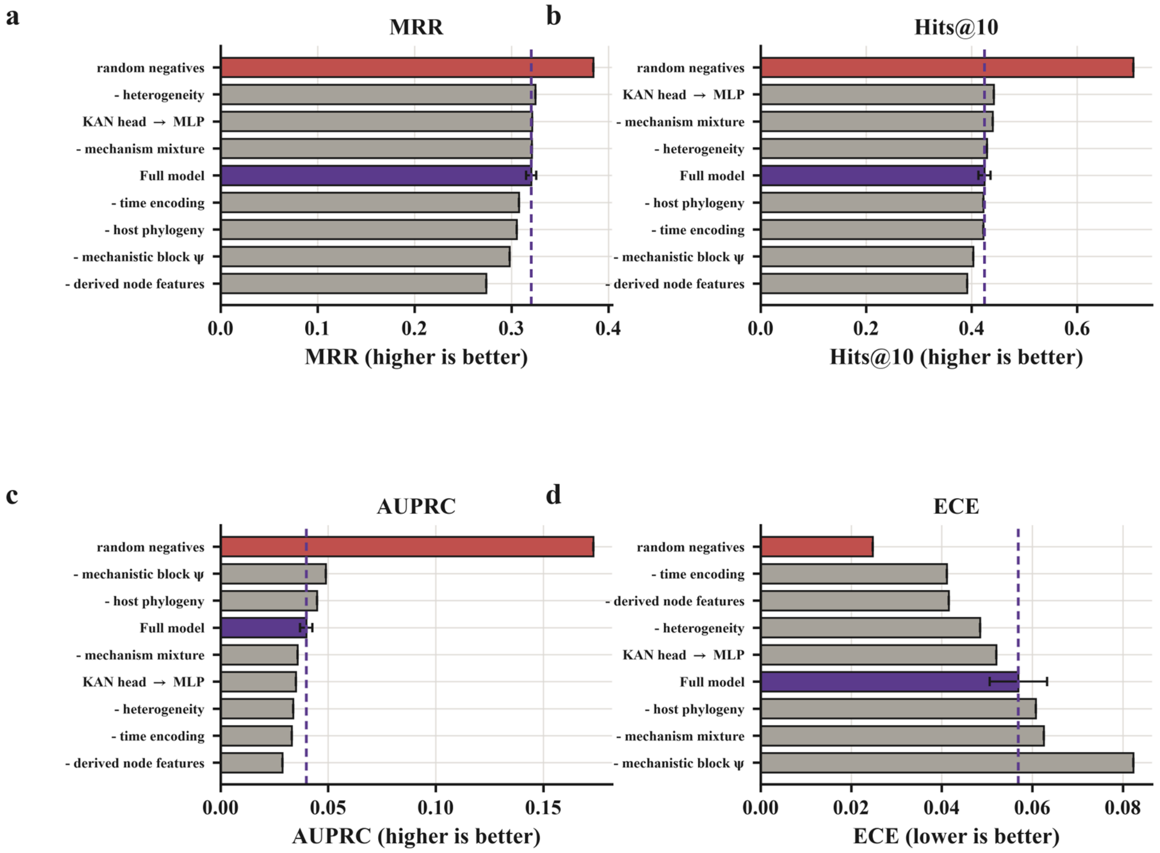

Figure S8. Ablation arms under the identical protocol, one panel per evaluation metric.

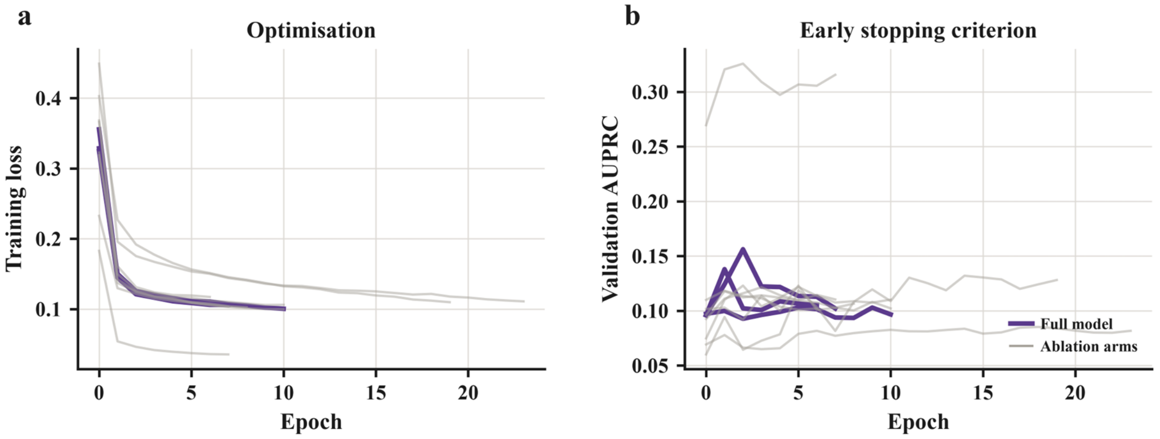

Figure S9. Training behaviour across arms. a Optimisation. b Early stopping criterion on validation precision-recall performance.

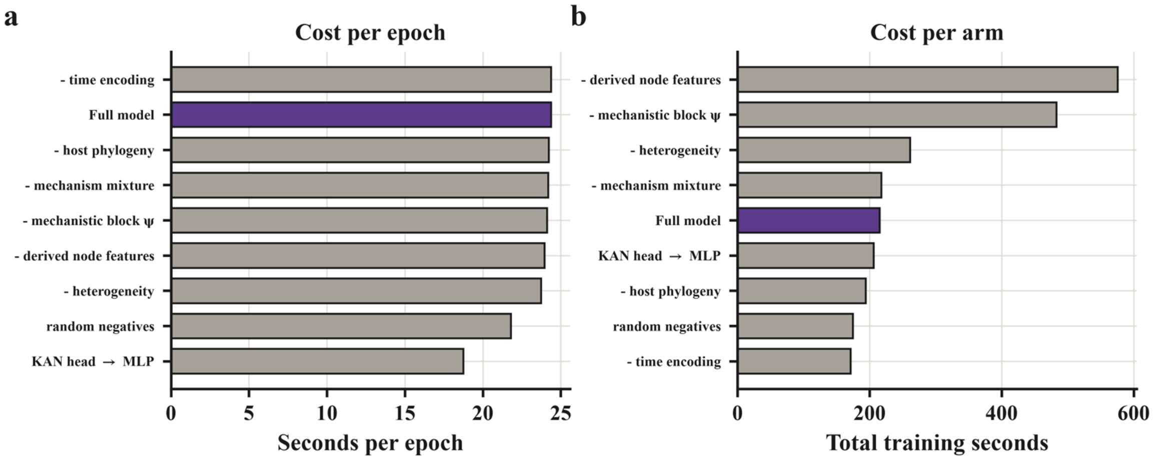

Figure S10. Computational cost. a Cost per epoch. b Total cost per arm.

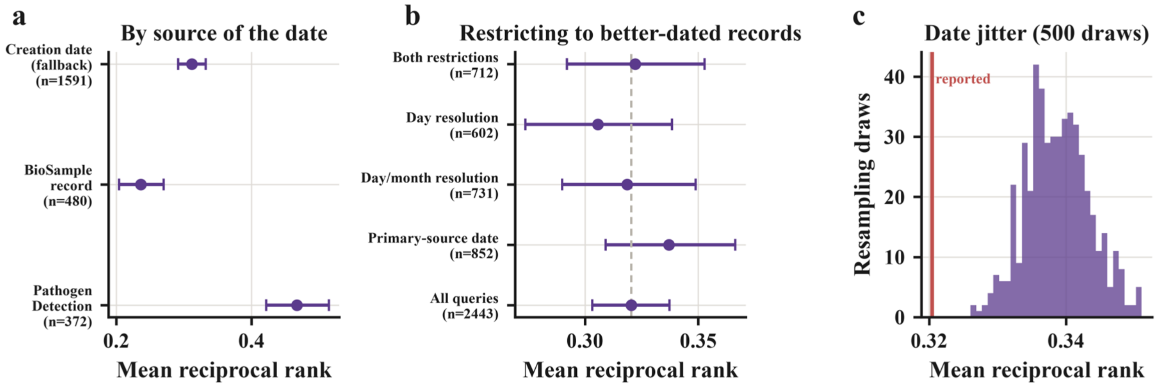

Figure S11. Sensitivity of the forecast to uncertainty in the reconstructed collection dates. a Mean reciprocal rank by the route that supplied the date, with bootstrap 95% confidence intervals. b The evaluation repeated on subsets restricted to better-resolved dates; the dashed line marks the pooled result. c Distribution of the mean reciprocal rank over 500 redrawings of every imprecise date, against the value reported in the main text.

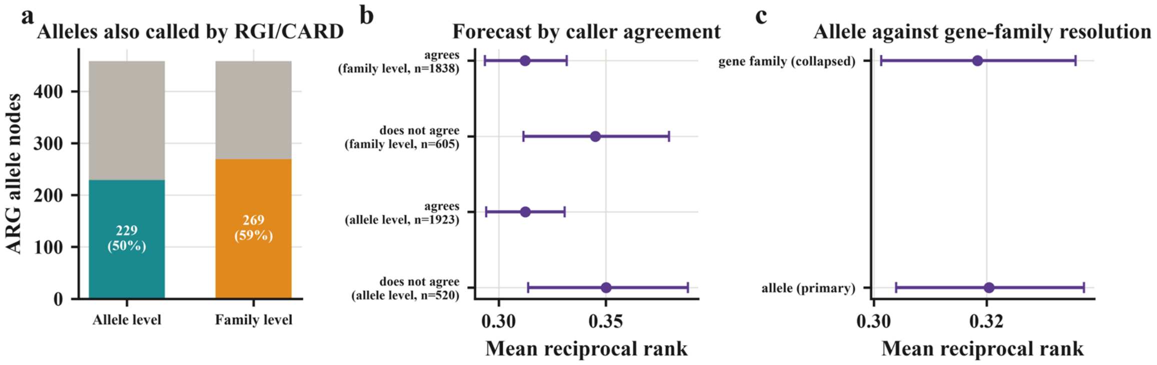

Figure S12. Robustness to the choice of resistance gene caller. a ARG allele nodes that also carry a call from the Resistance Gene Identifier, at allele and at gene-family level. b Mean reciprocal rank for queries whose gene the second caller does and does not report. c The evaluation repeated with the gene family in place of the allele as the unit of prediction.

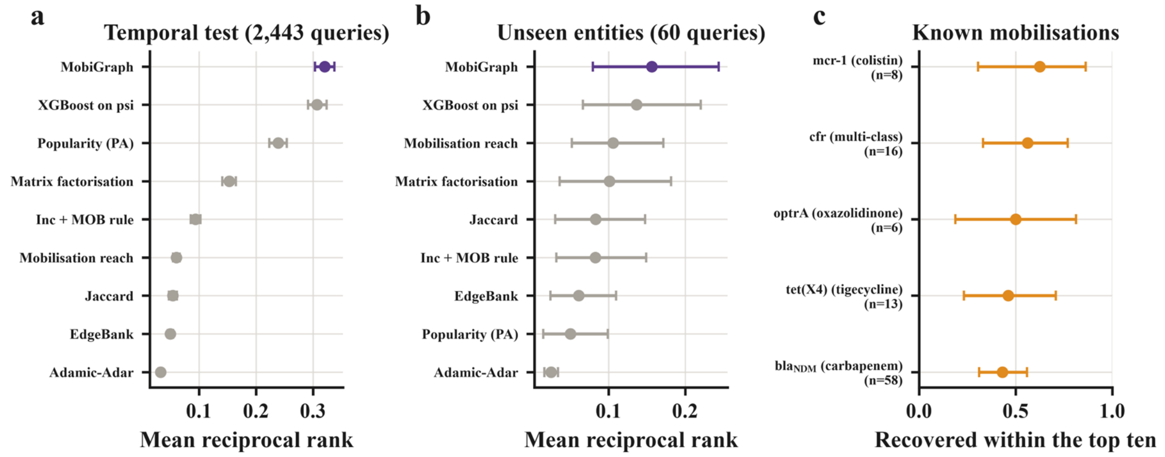

Figure S13. Uncertainty intervals on every method and stratum. a Percentile bootstrap intervals on the temporal test window. b The same for the 60 inductive queries, where the intervals of the leading methods overlap. c Wilson intervals on the fraction of post-cutoff events recovered within the top ten, for each documented mobilisation.

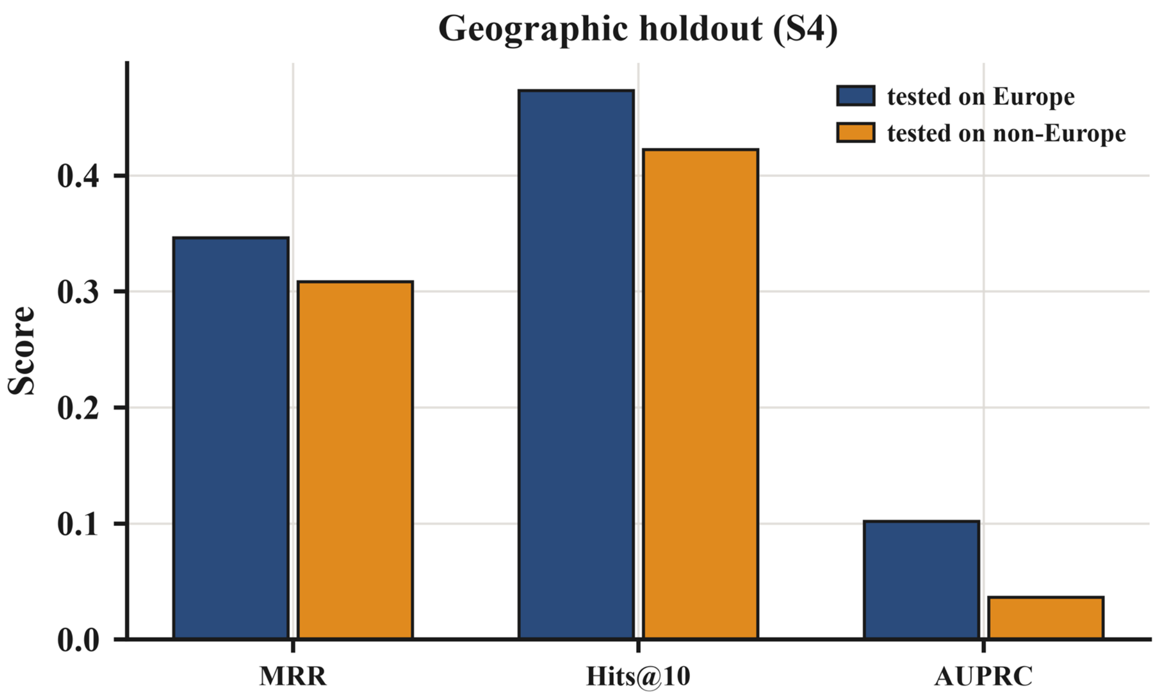

Figure S14. Geographic holdout in full, with every metric reported separately for triples first observed inside and outside Europe.

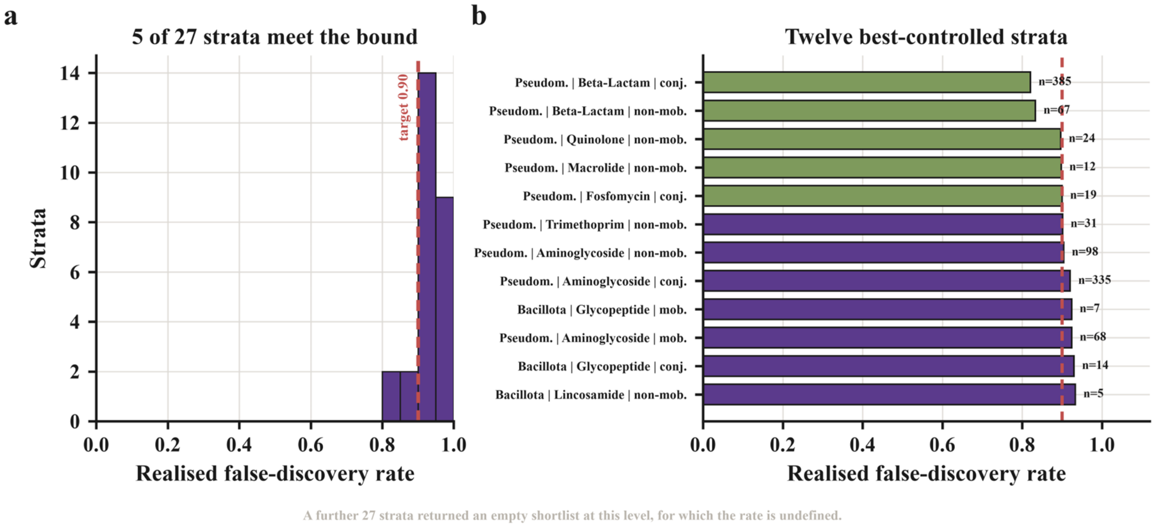

Figure S15. Stratified risk control. a Number of strata meeting the bound. b The twelve best-controlled strata.

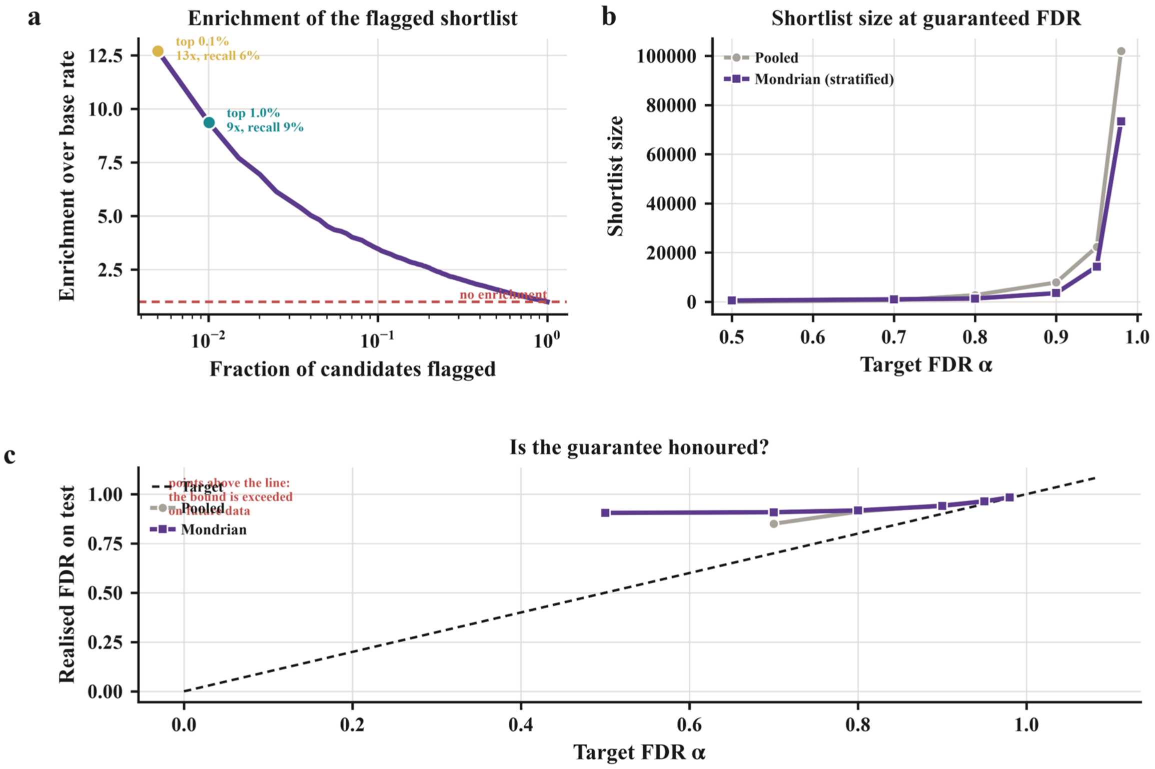

Figure S16. Operating characteristics of the conformal shortlist. a Enrichment over the base rate as the shortlist widens. b Shortlist size against the target level, pooled and stratified. c Realised against target level.

Supplementary Tables

Table S1. Glossary of the modelling terms used in the manuscript, for readers approaching the work from microbiology or epidemiology.

Term	Meaning
Link prediction	Scoring pairs or triples that a network does not yet join, for how likely they are to be joined next.
Node, edge	An entity in the graph (a gene allele, a replicon backbone, a host species) and a recorded association between two of them.
Reciprocal rank, MRR	One divided by the position at which the true host appears in the ranked candidate list; the mean reciprocal rank averages that over queries. Higher is better; 1.0 means the true host was always first.
Recall at depth k	The fraction of queries in which the true host appears among the k highest-scoring candidates.
AUPRC, AUROC	Threshold-free summaries of how well a score separates recorded from unrecorded candidates. AUPRC is the more informative of the two when recorded cases are rare, as they are here.
Effort-matched negatives	Alternative hosts chosen to resemble the true host in how heavily that taxon has been sequenced, so that a model cannot score well simply by naming well-studied organisms.
Temporal split	Training on associations first recorded before a chosen date and testing on those first recorded after it, rather than hiding edges at random from a finished network.
Inductive, cold start	A prediction in which the gene, the backbone or the host had not been seen at all before the cut-off.
Memory, temporal graph network	A vector held for each entity and updated whenever a new association involving it is recorded, so that the model carries a history rather than a snapshot.
Spline scoring head	A scoring function in which each input quantity has its own learned curve, so the contribution of each biological quantity can be read off and expressed as a rule.
Conformal prediction	A procedure that turns scores into a set carrying a stated error rate, without assuming a model for how the data were generated.
Covariate shift	The candidates proposed later differ systematically from those used to calibrate; weighted conformal prediction corrects for it by reweighting the calibration points.

False discovery proportion	The share of a returned shortlist that is not confirmed.
Mondrian calibration	Calibrating separately inside strata, here defined by host phylum, drug class and predicted mobility, so that one well-sampled group cannot carry the guarantee for the rest.
Lead time	The interval between the prediction cutoff and the first record of the predicted combination.
Incompatibility group	A classification of plasmid replicons; two plasmids of the same group are not stably maintained in the same cell.
Relaxase, oriT, MPF	Components of conjugative transfer: the relaxase nicks the origin of transfer (oriT) and the mating-pair formation system (MPF) builds the apparatus through which DNA passes.
MOB cluster, backbone	A group of near-identical plasmid sequences treated as one replicon entity, which is the level at which a surveillance programme acts.
Patristic distance	The distance between two species measured along the branches of a phylogenetic tree.

Table S2. Agreement between the two resistance gene callers applied to every plasmid record. Symbol conventions differ between the callers, so exact-symbol agreement is a lower bound.

Quantity	Value
Records with an AMRFinderPlus call	22734.000
Records with an RGI/CARD call	14492.000
Records scanned by both callers	14100.000
AMRFinderPlus calls on shared records	90382.000
RGI/CARD calls on shared records	36156.000
Calls agreeing at allele level	12773.000
Jaccard index, allele level	0.112
Calls agreeing at gene-family level	12952.000
Jaccard index, gene-family level	0.114
RGI/CARD calls recovered by AMRFinderPlus	0.353

Table S3. The fifteen resistance determinants most frequently recorded in the corpus, with the number of plasmid records, host species and replicon backbones on which each has been seen and the earliest collection date at which it appears.

Allele	Drug class	Records	Host species	Backbones	First recorded
sul1	SULFONAMIDE	5,305	120	244	1956-07-01
blaTEM-1	BETA-LACTAM	4,988	77	247	1970-07-01
sul2	SULFONAMIDE	4,024	105	197	1951-07-01
tet(A)	TETRACYCLINE	3,771	68	179	1971-07-01

aph(6)-Id	AMINOGLY-COSIDE	3,704	100	223	1969-07-01
aph(3'')-Ib	AMINOGLY-COSIDE	3,698	104	226	1969-07-01
mph(A)	MACROLIDE	2,677	71	147	1997-07-01
aadA2	AMINOGLY-COSIDE	2,366	70	164	1975-07-01
aadA1	AMINOGLY-COSIDE	2,136	82	200	1956-07-01
floR	PHENICOL	1,987	86	140	1984-10-14
aph(3')-Ia	AMINOGLY-COSIDE	1,825	70	161	1972-07-01
ble	BLEOMYCIN	1,790	80	122	1970-07-01
qnrS1	QUINOLONE	1,748	53	123	2002-01-15
aac(6')-Ib-cr5	AMINOGLY-CO-SIDE/QUINO-LONE	1,719	60	130	1984-10-14
dfrA12	TRIME-THOPRIM	1,669	46	122	1984-10-14

Table S4. Composition of the corpus by host phylum. Bacillota and Actinomycetota are the Gram-positive phyla represented.

Host phylum	Observations	Share	Species
Pseudomonadota	88,033	88.5%	144
Bacillota	11,181	11.2%	63
Campylobacterota	232	0.2%	2
Actinomycetota	42	0.0%	7
Bacteroidota	31	0.0%	5

Table S5. The same methods and the same positive examples scored against uniformly drawn negatives and against effort-matched negatives.

Method	AUPRC (emns)	AUPRC (random)	AUROC (emns)	AUROC (random)	MRR (emns)	MRR (random)
Adamic-Adar	0.0102	0.0152	0.5123	0.6381	0.0323	0.0483
EdgeBank	0.0125	0.0289	0.5977	0.7651	0.0497	0.1241
Mobilisation reach	0.0130	0.0328	0.6005	0.8113	0.0601	0.1337
Jaccard	0.0091	0.0112	0.4807	0.5684	0.0540	0.0321
Matrix factorisation	0.0132	0.0329	0.6153	0.7937	0.1529	0.2040
Popularity (PA)	0.0195	0.0922	0.6462	0.8469	0.2388	0.3100
Inc + MOB rule	0.0158	0.0434	0.6648	0.8359	0.0936	0.1367
XGBoost on psi	0.0428	0.1006	0.7336	0.8714	0.3071	0.3101

Table S6. Recall at the deeper positions of the candidate pool. Each pool holds one true host and 100 draws from the matched set, so recall at depth 100 approaches saturation for every method and carries little information about ranking quality.

Method	Recall@25	Recall@50	Recall@100
Popularity (PA)	0.5727	0.7749	0.9742
Adamic-Adar	0.0872	0.5305	0.9775
Jaccard	0.1105	0.2333	0.8936
Inc + MOB rule	0.3561	0.7921	0.9992
EdgeBank	0.2075	0.6934	0.9996
Mobilisation reach	0.2521	0.7929	0.9795
Matrix factorisation	0.3844	0.6799	0.9742
XGBoost on psi	0.6296	0.8178	0.9885
MobiGraph	0.6631	0.8518	0.9922

Table S7. Ranking metrics with percentile bootstrap 95% confidence intervals over queries, for every method on the temporal test window and on the inductive subset. MobiGraph entries are means across three seeds.

Split	Method	Queries	MRR (95% CI)	Recall@10 (95% CI)
Temporal test	MobiGraph	2,443	0.3204 (0.304–0.337)	0.4242 (0.405–0.444)
Temporal test	XGBoost on psi	2,443	0.3071 (0.291–0.324)	0.4147 (0.395–0.433)
Temporal test	Popularity (PA)	2,443	0.2388 (0.224–0.254)	0.3385 (0.320–0.357)
Temporal test	Matrix factorisation	2,443	0.1529 (0.141–0.165)	0.1985 (0.183–0.214)
Temporal test	Inc + MOB rule	2,443	0.0936 (0.085–0.102)	0.1343 (0.120–0.148)
Temporal test	Mobilisation reach	2,443	0.0601 (0.054–0.066)	0.0839 (0.072–0.095)
Temporal test	Jaccard	2,443	0.0540 (0.047–0.061)	0.0557 (0.046–0.065)
Temporal test	EdgeBank	2,443	0.0497 (0.045–0.055)	0.0679 (0.058–0.078)
Temporal test	Adamic-Adar	2,443	0.0323 (0.029–0.036)	0.0156 (0.011–0.020)
Unseen entities	MobiGraph	60	0.1563 (0.079–0.244)	0.1722 (0.083–0.272)
Unseen entities	XGBoost on psi	60	0.1363 (0.066–0.221)	0.1500 (0.067–0.250)
Unseen entities	Mobilisation reach	60	0.1056 (0.052–0.172)	0.1500 (0.067–0.250)
Unseen entities	Matrix factorisation	60	0.1010 (0.036–0.182)	0.1000 (0.033–0.183)
Unseen entities	Jaccard	60	0.0827 (0.030–0.148)	0.0833 (0.033–0.167)
Unseen entities	Inc + MOB rule	60	0.0825 (0.031–0.149)	0.0667 (0.017–0.133)

Unseen entities	EdgeBank	60	0.0609 (0.024–0.110)	0.1000 (0.033–0.183)
Unseen entities	Popularity (PA)	60	0.0498 (0.014–0.099)	0.0500 (0.000–0.100)
Unseen entities	Adamic-Adar	60	0.0244 (0.016–0.034)	0.0667 (0.017–0.133)

Table S8. Forecast performance under alternative treatments of collection-date uncertainty. Intervals are percentile bootstrap intervals over queries, except for the resampling row, where they are the 2.5th and 97.5th percentiles across draws. Values are means across three seeds.

Treatment	Queries	MRR (95% CI)	Recall@10
all test queries	2,443	0.3204 (0.303–0.337)	0.4242
date resolved from a primary source	852	0.3370 (0.309–0.366)	0.4624
day or month resolution	731	0.3186 (0.290–0.349)	0.4533
day resolution only	602	0.3057 (0.274–0.338)	0.4280
primary source and day/month resolution	712	0.3221 (0.292–0.353)	0.4583
date from Pathogen Detection	372	0.4669 (0.422–0.514)	0.6084
date from the BioSample record	480	0.2364 (0.204–0.270)	0.3493
creation-date fallback	1,591	0.3115 (0.291–0.332)	0.4037
500 redrawings of every imprecise date	973 per draw	0.3386 (0.330–0.348)	0.4560

Table S9. Agreement between the two resistance gene callers on the records both had scanned, and the support each ARG allele node in the graph receives from the second caller. The commonest unmatched calls are CARD entries that AMRFinderPlus does not report as acquired resistance determinants, together with alleles the two catalogues number differently.

Quantity	Value
Records scanned by both callers	14,100
Calls per record, AMRFinderPlus	6.7
Calls per record, Resistance Gene Identifier	2.6
Distinct calls, AMRFinderPlus	90,382
Distinct calls, Resistance Gene Identifier	36,156
Jaccard index, allele level	0.112
Jaccard index, gene-family level	0.114
RGI calls also made by AMRFinderPlus	35.3%
RGI calls matched when allele-level granularity is tolerated	51.0%
ARG allele nodes with an allele-level RGI call	229 of 458
ARG allele nodes with a family-level RGI call	269 of 458
Commonest unmatched RGI calls	mrx (2,796), aada (2,263), aac6ibcr6 (1,821), brpmbl (1,087), pc1betalactamaseblaz (1,006), catb3 (927), aac6ib10 (870), rmtb (846)

Table S10. Performance and calibration by host phylum. Strata holding fewer than 30 candidates or 5 positive cases are omitted.

Host phylum	Candidates	Positive cases	AUPRC	AUROC	ECE
Bacillota	14163	112	0.0852	0.8041	0.0362
Pseudomonadota	206924	2089	0.0412	0.7291	0.0437

Table S11. Size, yield and recall of the returned shortlist at each target level. Pooled calibration ignores stratum; Mondrian calibrates inside every host phylum by drug class by mobility cell.

alpha	lambda	feasible	min_achievable_fdr	shortlist_size	true_positives	realised_fdr	recall	precision	base_rate	lift	method	n_strata	n_fallback_strata
0.5000	0.9361	1.0000	0.0000	0	0	nan	0.0000	nan	0.0099	nan	pooled	nan	nan
0.5000	0.9361	1.0000	0.0000	0	0	nan	0.0000	nan	0.0099	nan	pooled_shift_corrected	nan	nan
0.5000	nan	nan	nan	516	49	0.9050	0.0201	0.0950	0.0099	9.5911	mondrian	195.0000	85.0000
0.5000	nan	nan	nan	681	67	0.9016	0.0274	0.0984	0.0099	9.9369	mondrian_shift_corrected	195.0000	85.0000
0.7000	0.7614	1.0000	0.0000	593	89	0.8499	0.0364	0.1501	0.0099	15.1585	pooled	nan	nan
0.7000	0.9361	1.0000	0.0000	0	0	nan	0.0000	nan	0.0099	nan	pooled_shift_corrected	nan	nan
0.7000	nan	nan	nan	992	91	0.9083	0.0372	0.0917	0.0099	9.2651	mondrian	195.0000	85.0000
0.7000	nan	nan	nan	910	92	0.8989	0.0377	0.1011	0.0099	10.2110	mondrian_shift_corrected	195.0000	85.0000
0.8000	0.6045	1.0000	0.0000	2733	243	0.9111	0.0995	0.0889	0.0099	8.9802	pooled	nan	nan
0.8000	0.9361	1.0000	0.0000	0	0	nan	0.0000	nan	0.0099	nan	pooled_shift_corrected	nan	nan

0.8000	nan	nan	nan	1319	109	0.9174	0.0446	0.0826	0.0099	8.3465	mondrian	195.0000	85.0000
0.8000	nan	nan	nan	1432	137	0.9043	0.0561	0.0957	0.0099	9.6627	mondrian_shift_corrected	195.0000	85.0000
0.9000	0.4010	1.0000	0.0000	7859	438	0.9443	0.1793	0.0557	0.0099	5.6290	pooled	nan	nan
0.9000	0.6045	1.0000	0.0000	2733	243	0.9111	0.0995	0.0889	0.0099	8.9802	pooled_shift_corrected	nan	nan
0.9000	nan	nan	nan	3525	210	0.9404	0.0860	0.0596	0.0099	6.0170	mondrian	195.0000	85.0000
0.9000	nan	nan	nan	3293	213	0.9353	0.0872	0.0647	0.0099	6.5329	mondrian_shift_corrected	195.0000	85.0000
0.9500	0.1581	1.0000	0.0000	22297	805	0.9639	0.3295	0.0361	0.0099	3.6465	pooled	nan	nan
0.9500	0.3088	1.0000	0.0000	11612	540	0.9535	0.2210	0.0465	0.0099	4.6969	pooled_shift_corrected	nan	nan
0.9500	nan	nan	nan	14302	517	0.9639	0.2116	0.0361	0.0099	3.6510	mondrian	195.0000	85.0000
0.9500	nan	nan	nan	7980	324	0.9594	0.1326	0.0406	0.0099	4.1008	mondrian_shift_corrected	195.0000	85.0000
0.9800	0.0078	1.0000	0.0000	101970	1776	0.9826	0.7270	0.0174	0.0099	1.7591	pooled	nan	nan
0.9800	0.0155	1.0000	0.0000	81780	1600	0.9804	0.6549	0.0196	0.0099	1.9760	pooled_shift_corrected	nan	nan
0.9800	nan	nan	nan	73351	1244	0.9830	0.5092	0.0170	0.0099	1.7129	mondrian	195.0000	85.0000
0.9800	nan	nan	nan	42623	810	0.9810	0.3316	0.0190	0.0099	1.9194	mondrian_shift_corrected	195.0000	85.0000

Table S12. Threshold rules read from the pruned scoring head, each selected on the training window and re-scored as a standalone classifier on the temporal test window. Thresholds are given in the original units of each feature.

Rule	Support (train)	Precision (train)	Lift (train)	Support (test)	Precision (test)	Lift (test)	Survives
host is new for this gene <= 0.5 AND log deposition volume for the host <= 2.48 AND number of hosts already carrying the gene <= 7	168	0.39	8.12	7	0.57	12.00	no
log co-occurrence of the gene family with this Inc group >= 6.15 AND log co-occurrence of the gene family with this host genus >= 6.54 AND log deposition volume for the host >= 8.89	2,725	0.29	6.16	229	0.19	4.03	yes
host is new for this gene <= 0.5 AND log deposition volume for the host <= 4.26 AND number of hosts already carrying the gene <= 7	887	0.28	5.94	50	0.18	3.78	yes
host is new for this gene <= 0.5 AND log co-occurrence of	730	0.28	5.90	93	0.27	5.65	yes

the gene family with this host genus >= 6.54 AND number of hosts already carrying the gene <= 7							
log co-occurrence of the gene family with this Inc group >= 4.93 AND log co-occurrence of the gene family with this host genus >= 6.54 AND log deposition volume for the host >= 8.89	4,755	0.28	5.78	448	0.18	3.70	yes
host is new for this gene <= 0.5 AND log co-occurrence of the gene family with this host genus >= 4.71 AND number of hosts already carrying the gene <= 7	1,951	0.27	5.72	224	0.22	4.69	yes
kb_to_oriT >= 500 AND log co-occurrence of the gene family with this host genus >= 6.54 AND log deposition volume for	3,090	0.27	5.67	449	0.16	3.37	yes

the host >= 8.89							
kb_to_oriT >= 190 AND log co-occurrence of the gene family with this host genus >= 6.54 AND log deposition volume for the host >= 8.89	3,234	0.27	5.60	462	0.16	3.36	yes
log co-occurrence of the gene family with this host genus >= 6.54 AND log deposition volume for the host >= 8.89 AND number of hosts already carrying the gene <= 39	3,037	0.27	5.59	437	0.18	3.80	yes
host is new for this gene <= 0.5 AND log co-occurrence of the gene family with this host genus >= 6.54 AND log deposition volume for the host >= 8.89	8,035	0.27	5.58	946	0.15	3.11	yes
log co-occurrence of the gene family with this Inc group >= 4.04 AND log co-	6,424	0.27	5.58	670	0.16	3.32	yes

occurrence of the gene family with this host genus >= 6.54 AND log deposition volume for the host >= 8.89

Table S13. Lead time between the training cutoff and first observation, by drug class, for triples reaching the top ten of their pool.

n	miss_rate	median_months	q25_months	q75_months	frac_positive_lead	drug_class	n_events	k
256	0.56	23.61	17.02	29.96	1.00	AMINOGLYCOSIDE	584	10
18	0.67	19.11	16.39	24.32	1.00	AMINOGLYCOSIDE/QUINOLONE	55	10
24	0.57	22.06	16.88	30.04	1.00	RIFAMYCIN	56	10
247	0.58	22.51	17.00	31.87	1.00	BETA-LACTAM	592	10
19	0.63	28.42	17.69	31.41	1.00	BLEOMYCIN	51	10
68	0.55	24.00	17.73	27.17	1.00	PHENICOL	151	10
86	0.54	22.70	16.57	29.96	1.00	TRIMETHOPRIM	188	10
37	0.61	25.66	16.59	29.83	1.00	MACROLIDE	94	10
16	0.27	19.22	16.81	29.61	1.00	LINCOSAMIDE/MACROLIDE/STREPTOGRAMIN	22	10
10	0.57	22.14	18.16	30.66	1.00	FOSFOMYCIN	23	10
10	0.52	24.76	16.93	30.53	1.00	LINCOSAMIDE	21	10
10	0.50	22.39	18.48	33.70	1.00	COLISTIN	20	10
8	0.69	30.01	23.74	35.33	1.00	MACROLIDE/STREPTOGRAMIN	26	10

64	0.56	21.32	16.99	30.04	1.00	QUINO-LONE	146	10
57	0.64	20.86	16.99	29.73	1.00	SULFONA-MIDE	159	10
78	0.56	19.71	16.43	29.91	1.00	TETRACY-CLINE	176	10
15	0.29	19.32	19.32	29.96	1.00	GLYCO-PEPTIDE	21	10

Table S14. Recovery of post-cutoff events for each documented mobilisation, with Wilson 95% confidence intervals. Event counts are small and the intervals are correspondingly wide.

Determinant	Events	Depth	Recovered	Fraction	95% CI
mcr-1 (colistin)	8	top 1	3	37.5%	0.14–0.69
mcr-1 (colistin)	8	top 5	3	37.5%	0.14–0.69
mcr-1 (colistin)	8	top 10	5	62.5%	0.31–0.86
bla_NDM (car-bapenem)	58	top 1	15	25.9%	0.16–0.38
bla_NDM (car-bapenem)	58	top 5	17	29.3%	0.19–0.42
bla_NDM (car-bapenem)	58	top 10	25	43.1%	0.31–0.56
tet(X4) (tigecy-cline)	13	top 1	5	38.5%	0.18–0.64
tet(X4) (tigecy-cline)	13	top 5	5	38.5%	0.18–0.64
tet(X4) (tigecy-cline)	13	top 10	6	46.2%	0.23–0.71
optrA (oxazoli-dinone)	6	top 1	2	33.3%	0.10–0.70
optrA (oxazoli-dinone)	6	top 5	2	33.3%	0.10–0.70
optrA (oxazoli-dinone)	6	top 10	3	50.0%	0.19–0.81
cfr (multi-class)	16	top 1	7	43.8%	0.23–0.67
cfr (multi-class)	16	top 5	7	43.8%	0.23–0.67
cfr (multi-class)	16	top 10	9	56.2%	0.33–0.77

Table S15. Confirmation of the frozen watch list with Wilson 95% confidence intervals, one-sided binomial tests against the background rate of 8.18%, and one-sided Fisher exact tests against the full scored candidate set. Confirmation is at gene–host resolution.

List depth	Candidates	Confirmed	Fraction (95% CI)	Enrichment	Binomial p	Fisher p
10	10	3	0.300 (0.108–0.603)	3.67	0.0424	0.0424
50	50	7	0.140 (0.070–0.262)	1.71	0.111	0.111
100	100	13	0.130 (0.078–0.210)	1.59	0.0644	0.0644
500	500	65	0.130 (0.103–0.162)	1.59	0.000161	0.000159
conformal shortlist	31	6	0.194 (0.092–0.363)	2.37	0.0373	0.0373
